# Using high-density SNP data to unravel the origin of the Franches-Montagnes horse breed

**DOI:** 10.1186/s12711-024-00922-6

**Published:** 2024-07-10

**Authors:** Annik Imogen Gmel, Sofia Mikko, Anne Ricard, Brandon D. Velie, Vinzenz Gerber, Natasha Anne Hamilton, Markus Neuditschko

**Affiliations:** 1 Animal GenoPhenomics, Agroscope, Route de la Tioleyre 4, 1725 Posieux, Switzerland; 2 Equine Department, Vetsuisse Faculty, University of Zurich, Winterthurerstrasse 260, 8053 Zurich, Switzerland; 3 Department of Animal Breeding and Genetics, Swedish University of Agricultural Sciences, Box 7023, 750 07 Uppsala, Sweden; 4 Institut National de la Recherche Agronomique, Domaine de Vilvert, 78350 Jouy-en-Josas, France; 5 Equine Genetics and Genomics Group, School of Life and Environmental Sciences, University of Sydney, RMC Gunn B19-603, Sydney, NSW 2006 Australia; 6 Institut Suisse de Médecine Equine ISME, Vetsuisse Faculty, University of Bern, Länggassstrasse 124, 3012 Bern, Switzerland; 7 Sydney School of Veterinary Science, University of Sydney, Sydney, NSW 2006 Australia

## Abstract

**Background:**

The Franches-Montagnes (FM) is the last native horse breed of Switzerland, established at the end of the 19th century by cross-breeding local mares with Anglo-Norman stallions. We collected high-density SNP genotype data (Axiom™ 670 K Equine genotyping array) from 522 FM horses, including 44 old-type horses (OF), 514 European Warmblood horses (WB) from Sweden and Switzerland (including a stallion used for cross-breeding in 1990), 136 purebred Arabians (AR), 32 Shagya Arabians (SA), and 64 Thoroughbred (TB) horses, as introgressed WB stallions showed TB origin in their pedigrees. The aim of the study was to ascertain fine-scale population structures of the FM breed, including estimation of individual admixture levels and genomic inbreeding (F_ROH_) by means of Runs of Homozygosity.

**Results:**

To assess fine-scale population structures within the FM breed, we applied a three-step approach, which combined admixture, genetic contribution, and F_ROH_ of individuals into a high-resolution network visualization. Based on this approach, we were able to demonstrate that population substructures, as detected by model-based clustering, can be either associated with a different genetic origin or with the progeny of most influential sires. Within the FM breed, admixed horses explained most of the genetic variance of the current breeding population, while OF horses only accounted for a small proportion of the variance. Furthermore, we illustrated that FM horses showed high TB admixture levels and we identified inconsistencies in the origin of FM horses descending from the Arabian stallion Doktryner. With the exception of WB, FM horses were less inbred compared to the other breeds. However, the relatively few but long ROH segments suggested diversity loss in both FM subpopulations. Genes located in FM- and OF-specific ROH islands had known functions involved in conformation and behaviour, two traits that are highly valued by breeders.

**Conclusions:**

The FM remains the last native Swiss breed, clearly distinguishable from other historically introgressed breeds, but it suffered bottlenecks due to intensive selection of stallions, restrictive mating choices based on arbitrary definitions of pure breeding, and selection of rare coat colours. To preserve the genetic diversity of FM horses, future conservation managements strategies should involve a well-balanced selection of stallions (e.g., by integrating OF stallions in the FM breeding population) and avoid selection for rare coat colours.

**Supplementary Information:**

The online version contains supplementary material available at 10.1186/s12711-024-00922-6.

## Background

The Franches-Montagnes (FM) is the last native horse breed of Switzerland, established at the end of the 19th century from crossbreeding local Jura and Comtois mares with two Anglo-Norman founding sires, Vaillant (1891) and Imprévu (1886). The FM studbook was officially released in 1921 [1, 2]. Periodic introgression of horses from other breeds were allowed to meet market demands, initially for heavier draught horses required for agricultural work, which led to cross-breeding with Percheron, Breton, and Belgian draught horses [1, 2]. In response to the increased mechanisation of agriculture after World War II, additional introgressions using Shagya Arabian, Purebred Arabian, Nonius, Anglo-Norman, Selle Français, and Swiss Warmblood stallions were carried out to select a lighter horse type for leisure riding and carriage driving. Some of these introgressions also led to the formation of new sire lines. Traditionally, the sire lines of the FM breed are expressed as letters, where each stallion’s name starts with the same letter as his  sire. Currently, there are 11 extant sire lines: C, D, E, H, R, and V are considered original, although the most influential V sire (Vulcain, 1983) had a Warmblood damsire. Additional lines, such as the DON, L, N, P, and Q lines, descend from sires used for cross-breeding. The DON line originated from the purebred Arabian stallion Doktryner (1950), which covered FM mares from 1958, with only one of his sons (Don Pablo, 1964) producing licenced stallions himself. In the 1970s, the Swedish Warmblood stallions Aladin (1964) and Nello (1971), sons of the Trakhener stallion Nepal (1956) were selected, resulting in the sire lines L and P. The last introgressions involved two Swiss Warmblood stallions from the same dam (Salomé, 1977), Qui-Sait (1985) and Noé (1984), establishing the sire lines Q and N, respectively.

To preserve the breed characteristics obtained during its admixed history, the studbook was closed in 1997. To date, admixture proportions are estimated based on pedigree information as a total percentage, while breed origin is ignored. Furthermore, by convention, FM horses born before 1950 are considered purebred, meaning that earlier introgressions are not accounted for in the current computation of admixture proportions. Since 2012, purebred or old-type FM (hereafter, OF and considered as a separate breed) are managed as a subsection of the FM studbook. The OF selection criteria (field test, stallion licensing) differ from those of the main FM population. The main breeding goal of the OF is to maintain a heavier draught horse, while the focus of the FM is to breed a leisure horse for riding and carriage driving. Both FM and OF breeders share a common goal of preserving the perceived good temperament of the breed. Currently, OF is declining in numbers, resulting in increased inbreeding in this subpopulation. Conversely, additional cross-breeding activities threaten the integrity of the FM horse population as an original native breed, both from a genetic [3] and a phenotypic perspective [4].

Runs of homozygosity (ROH) in an individual’s genome, which consist of haplotypes identical-by-descent (IBD), are one of the main tools to quantify genomic levels of inbreeding. The length of ROH segments can also be used to ascertain historical changes in population size and structure. Recombination and admixture events are major reasons for breakdown of ROH, such that long ROH segments indicate recent inbreeding, while the genome-wide assessment of ROH segments has emerged as a valuable tool to estimate inbreeding. Hence, outcrossed animals typically carry fewer and shorter ROH segments, while animals descending from long-term inbred populations carry more and shorter ROH segments. Recently inbred animals (e.g. bottleneck) carry fewer and longer ROH segments (see Ceballos et al. [5] for a complete review). The genomic inbreeding coefficient (F_ROH_) for an animal is derived by dividing the sum of all homozygous segments (S_ROH_) by the total length of the genome [6]. Numerous studies have demonstrated that same overlapping ROH segments between multiple individuals, so-called ROH islands, also highlight genetic regions that have been under selection, in cattle [7, 8], sheep [9–11], and honeybees [12]. In horses, based on different genotyping platforms and ROH settings, numerous breed-specific ROH islands have already been identified that include genes associated with behaviour [13, 14], coat colour [14–18], coat quality [19], fertility [13, 18–22], size [15, 22], skeletal development [18, 19, 23], muscle growth [24], geographical adaptations [14, 25], and gait quality [14].

In this study, we used high-density SNP genotypes from the Affymetrix Axiom^™^ Equine genotyping array to ascertain the fine-scale population structure of the FM breed, taking into account the last four cross-breeding activities. Furthermore, we determined the level of F_ROH_ in all breeds by means of ROH. Within FM we additionally compared F_ROH_ against pedigree-based inbreeding (F_PED_) and admixture proportions. Finally, we identified private and overlapping ROH islands to determine genomic regions that are under selection in the respective breed.

## Methods

### Dataset

The genomic data consisted of 1268 horses originating from five different European breeds. We collected 44 samples of OF and 478 of FM. Between 2014 and 2023, we genotyped nearly all three-year old males selected for the stallion licensing test, since genetic testing for congenital hepatic fibrosis became mandatory in 2012 [26]. The DNA from older stallions was available through frozen sperm, including  two F1 sires that descended from the Warmblood stallions Noé and Qui-Sait. Additional geldings and mares were genotyped in the same period of time, such that all 11 extant sire lines were represented by eight (Q line) up to 148 descendants (H line).

Furthermore, we included 32 Shagya Arabians (SA) because of the Shagya introgression in the 1940s, 136 Purebred Arabians (AR) descending from the introgressed stallion Doktryner at the 5th to 8th generation, and 514 European Warmblood horses (WB, 380 belonging to the Swedish Warmblood Studbook, and 134 to the Swiss Warmblood Studbook). The WB samples included one of the two Swiss Warmblood stallions (Noé, 1984) that was used for cross-breeding in the 1990s. Finally, we included a convenience sample of 64 Thoroughbred horses (TB) from Australasia, as the WB stallions used for cross-breeding also showed TB origin in their pedigrees.

The DNA from FM and Swiss WB was sampled under permits VD3527b, VD2976.1, and VD2227.2, each approved by the cantonal veterinary office of Vaud, Switzerland, while the genotype information from the other breeds was derived from previous studies [13, 14].

### SNP genotyping

All 1268 horses were genotyped on the commercial Axiom™ Equine Genotyping array that contained 670,796 evenly distributed single nucleotide polymorphisms (SNPs) [27]. The chromosomal position of the SNPs was determined based on the EquCab3.0 reference genome [28] and the most recent annotation file (https://www.thermofisher.com/order/catalog/product/550583). Single nucleotide polymorphisms positioned on the sex chromosomes or with unknown chromosomal position were excluded from further analyses, resulting in 602,131 SNPs. Missing genotype information was imputed separately for each breed using BEAGLE 5.2 [29]. For population clustering and admixture analysis, we excluded SNPs with a minor allele frequency (MAF) less than 0.05, resulting in 421,988 SNPs per horse.

### Population clustering

To illustrate the population structure of the horses, we performed a principal component analysis (PCA) and computed pairwise F_ST_ distances between the breeds. Principal Component Analysis was applied to a genetic relationship matrix (*G*) with pairwise identities by state (IBS) between horses, as provided by PLINK v1.9 [30], and the first two principal components (PCs) were visualized using the R-platform (www.r-project.org) [31]. We also calculated pairwise F_ST_ estimates between breeds using the Wright’s F_ST_ function, as implemented in PLINK v 1.9 [30], and used the program SPLITSTREE (www.splistree.org) [32] to create phylogenetic networks between the breeds.

### Admixture

Admixture proportions for each horse were determined using the program Admixture 1.23 [33]. We ran Admixture for 100 iterations, increasing K from 2 to 20. Convergence between independent runs at the same K was monitored by comparing the resulting log-likelihood scores (LLs) following 100 iterations and was inferred from stabilized LLs with less than 1 LL unit of difference between runs. Cross validation (CV) error estimation for each K was performed to determine the optimal number of clusters. We also derived the admixture proportions of FM horses using the supervised learning mode, by specifying the ancestries of the reference individuals according to the five sampled breeds (TB, AR, SA, WB and OF). Supervised (K = 5) and unsupervised admixture results by increasing K from 2 to 10, were visualized with the program Distruct 1.1 [34] and integrated in the high-resolution FM population network, as described below.

### Identification of key contributors

Within the FM breed, in addition to the admixture proportions, we also identified key contributors to ascertain the impact of admixed horses on the current population. The method to identify key contributors which explain most of the genetic variance in complex population structures was described in detail by Neuditschko et al. [35] and available online at https://github.com/esteinig/netview. It requires as input a symmetric relationship matrix *A* of dimension *n* × *n* and the number of *k* significant PCs, where *n* is the number of individuals. Here, we used the aforementioned IBS relationship matrix *G* and determined the number of *k* significant PCs with the empirical method described as Horn’s parallel analysis, which is implemented in the statistical software package paran [36]. This method employs Monte Carlo estimates to retain the most significant PCs under a defined level of significance and number of iterations. Here, we chose a significance level of P = 0.01 and 10,000 iterations, which have been suggested in the modified version of Horn’s parallel analysis [37]. After determining the number of *k* significant PCs, we calculated the genetic contribution score (*gc*_*j*_) for each horse. The computation of the *gc*_*j*_ is based on a Singular Value Decomposition (SVD) of *G* and accounts for the correlation between the *j*th individual relationship vector and the *i*th standardized eigenvector, limiting the number of eigenvectors to the first *k* significant PCs. Thereafter, we ranked the horses according to *gc*_*j*_ and also explored the association between *gc*_*j*_ and individual admixture proportions.

### High-resolution population networks

A high-resolution population network visualization of FM horses was performed by applying NetView, as described in detail by Neuditschko et al. [38] and Steinig et al. [39]. Briefly, we computed genetic distances by subtracting pairwise IBS relationships from 1 and applied the algorithm in its default setting (number of k nearest neighbours k-NN = 10). To illustrate the genetic relatedness between neighbouring horses, we associated the thickness of edges (connecting lines) with the magnitude of the genetic distance, with thicker edges corresponding to lower genetic distances. To identify highly influential and inbred horses within the respective population networks, we scaled the node size of each horse based on the individual *gc*_*j*_ and F_ROH_ (see computation below), with the node colour representing the individual admixture proportions at the respective number of clusters (unsupervised K = 7 and supervised K = 5).

### Runs of homozygosity

Runs of homozygosity segments were determined with an overlapping window approach implemented in PLINK v1.9 [30], using all 602,131 genome-wide mapped SNPs. The following settings were applied: a minimum SNP density of one SNP per 50 kb, a maximum gap length of 100 kb, and a minimum length of homozygous segments of 500 kb (including more than 80 homozygous SNP genotypes), while one heterozygous SNP genotype was permitted per segment. The total number of ROH (N_ROH_), the total length of ROH segments (S_ROH_), and the average length of ROH (L_ROH_) were summarised for each breed. The genomic inbreeding coefficients (F_ROH_) were calculated by dividing S_ROH_ by the length of the autosomal genome (L_AUTO_), which was set to 2280.92 Mb. Differences between breeds were investigated using ANOVA and post hoc Tukey’s HSD (honestly significant difference) tests at a significance level of α < 0.05, as implemented in the R package multcompView [40]. The length of segments was also categorised for each breed to explore breed-specific ROH islands. We also compared F_ROH_ and cumulative genomic admixture proportions (A_GEN_) of each FM horse with their respective pedigree-based inbreeding (F_PED_) and admixture proportions (A_PED_), using a linear regression model, as implemented in the statistical computing software R [31]. The cumulative genomic admixture proportions were provided by the FM association, while F_PED_ of the horses was calculated with the R package pedigree [41].

### Runs of homozygosity islands and gene functions

Putative ROH islands were determined based on overlapping homozygous regions that were shared by more than 50% of horses within each breed, using the R package detectRUNS [42]. Runs of homozygosity islands that occurred in only one breed were defined as “breed-specific” or “private”. Additionally, we also determined overlapping ROH islands between breeds, using a threshold for the minimal overlap of at least two SNPs. We used the NCBI genome data viewer (https://www.ncbi.nlm.nih.gov/genome/gdv/) and the reference genome assembly EquCab3.0 [28] to identify genes located in ROH islands. Gene ontology (GO) analysis on the identified genes was performed for each breed using the term enrichment service provided on https://amigo.geneontology.org/amigo, with the settings reference list “Equus caballus” and Fisher’s Exact test with Bonferroni correction for multiple testing. The gene list was limited to uniquely mapped gene IDs. Known functions of the identified genes were assigned  by conducting a literature review and allele frequencies of functional SNPs were calculated for each breed, i.e. SNPs with known effects on specific phenotypes.

## Results

### Population clustering

**Fig. 1:**
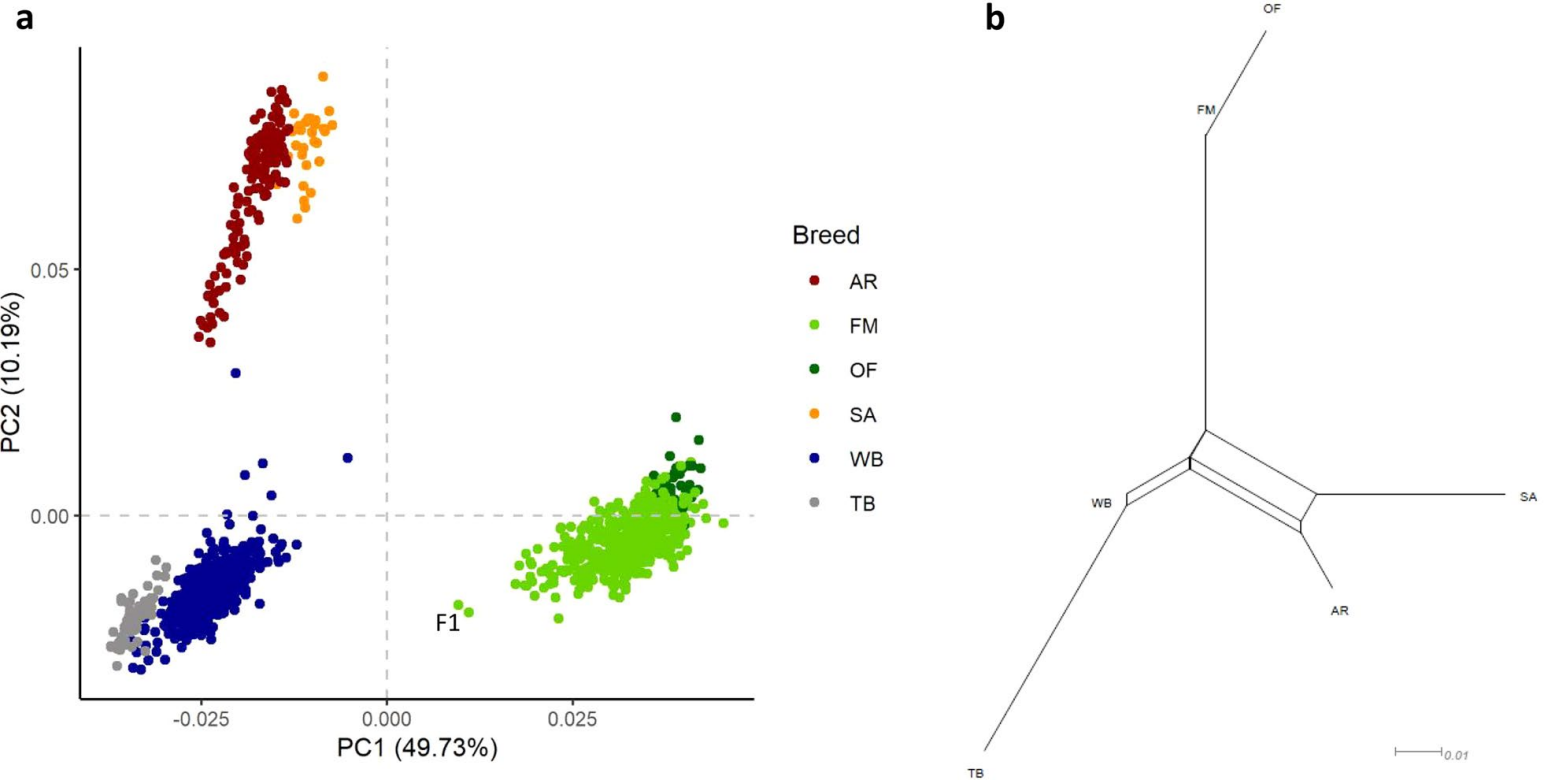
Population clustering of the six horse breeds. **a** Visualization of horses on the first two principal components (PCs) explaining 60% of the genetic variance; and **b** phylogenetic network of pairwise F_ST_ distances between breeds and subpopulations

Visualization of the first versus the second PC showed that the six breeds comprise three distinct population clusters (Fig. 1a). The first PC, accounting for 50% of the genetic variance, differentiated OF and FM from the other breeds, while the second PC, accounting for 10% of the genetic variance, separated the two Arabian breeds (AR and SA) from WB and TB. Based on the first two PCs, it was not possible to clearly distinguish the two Arabian breeds from each other, i.e. WB from TB and OF from FM horses. The two F1 crosses showed a noticeable distance to the remaining FM horses, while the WB sire that was used for cross-breeding was not positioned next to the F1 crosses. The topology of the phylogenetic network based on F_ST_ distances coincided with the visualization of the first two PCs, thereby refining the genetic distances between breeds (Fig. 1b). The lowest F_ST_ distance between breeds was observed for WB and TB (0.05), followed by SA and AR (0.06). Old-type FM horses showed the highest F_ST_ distances to all breeds, in the following decreasing order TB (0.19), SA (0.16), AR (0.14), WB (0.11). Compared to OF, pairwise F_ST_ distances between FM and the other breeds were slightly lower. The largest differences between OF and FM pairwise F_ST_ distances were observed for TB (0.04), followed by SA (0.03), while the distance between the two subpopulations, FM and OF, was 0.02.

### Admixture

**Fig. 2:**
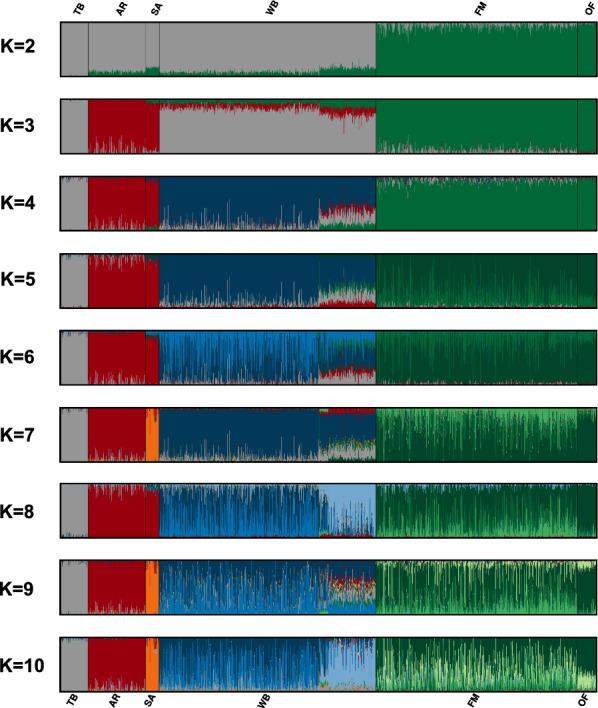
Model-based clustering result. Graphical representation of individual cluster membership coefficients of horses increasing the number of clusters (K) from 2 to 10

While increasing K from 1 to 20, the CV error estimation did not provide an optimal cluster solution (see Additional file 1: Figure S1). The first level of model-based clustering (K = 2) confirmed the findings of the first PC, by allocating FM and OF horses into a distinct population cluster and simultaneously pinpointed admixed FM horses, while OF horses were less admixed (Fig. 2). At K = 3, the two Arabian breeds (AR and SA) formed a distinct cluster, whereas some AR horses showed a high level of admixture with TB horses. At the next level (K = 4), WB horses were differentiated from TB horses, although all WB horses showed high TB admixture levels. At two additional level of clustering, FM (K = 5) and WB (K = 6), horses were sub-structured, before SA were differentiated from AR horses at K = 7. Hence, we considered K = 7 to be an optimal cluster solution, as all sampled breeds built a distinct population cluster. Further, when increasing K to 10, in particular WB and FM horses were further sub-structured, while at K = 8 and K = 10, WB horses from Switzerland were allocated to a distinct population cluster (light blue), although these horses already showed a different admixture pattern at all previous levels of clustering (e.g., at K = 4 WB horses from Switzerland had higher TB and Arabian admixture levels compared to WB horses from Sweden).

**Fig. 3:**
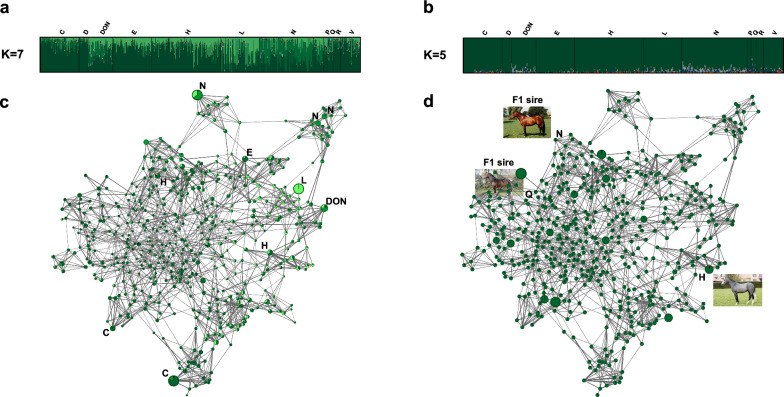
Admixture results and high-resolution population network visualizations of FM horses. **a** Membership coefficients of FM horses, ordered along stallion line, at unsupervised clustering K = 7; **b** membership coefficients of FM horses, ordered along stallion line, at supervised clustering K = 5; **c** high resolution population network, where each FM horse is illustrated by a node, with individual node size proportional to ${gc}_{j}$, while the node color represents the individual levels of admixture at unsupervised K = 7. The thickness of edges varies in the proportion of the genetic distance to visualize individual relationships between the horses. Top key contributors are highlighted by their respective stallion line; and **d** high resolution population network, where each FM horse is illustrated by a node, with individual node size proportional to F_ROH_, while the node colour represents the individual levels of admixture at supervised K = 7. The two F1 sires and the highly inbred grey horse are highlighted by images and their respective stallion line

Re-ordering the cluster membership proportions of FM horses at K = 7 according to the 11 sire lines (Fig. 3a) also revealed that the FM substructures were associated with a different genetic background, as especially horses that descended from the introgressed stallions Noé (sire line N) and Aladin (sire line L) were assigned to distinct population clusters, while the substructure associated with sire line N (last introgression) was already apparent at K = 5 (Fig. 2). However, it should be noticed, that the admixture profile of FM horses at K = 7 was poorly associated with sire line origin, as horses that originated from a different sire line showed the same admixture pattern and vice versa. Applying the supervised learning mode at K = 5, admixed FM horses were identified across all sire lines, while especially horses from sire lines DON, L, N, and Q showed high TB and WB admixture levels (Fig. 3b). In this context, it was not expected that horses descending from the Arabian stallion Doktryner (line DON) had higher TB than AR admixture levels, which were similar to those observed for horses descending from the Warmblood stallion Noé (line N). All admixed FM horses displayed unanticipated high TB admixture levels, as no TB stallions were used for cross-breeding in the FM breeding history.

### Identification of key contributors and high-resolution network visualization

Applying Horn’s parallel analysis on $G$ including only FM horses resulted in 33 significant PCs that accounted for 78% of the genetic variance (result not shown). After determining ${gc}_{j}$ and F_ROH_ for each horse, we performed two high-resolution network visualizations (Fig. 3c, d). The network visualization that illustrates ${gc}_{j}$ and individual admixture levels of each horse at unsupervised clustering K = 7, represented a common observed topology, where key contributors and their respective progeny built distinct population clusters at the edge of the network (Fig. 3c). Simultaneously, it was noted that some of these population clusters caused the aforementioned substructures in the FM population. Ranking the horses according to ${gc}_{j}$ showed that the F1 sire Népal was amongst top key contributors (ranked on 11th position), while the other F1 sire Queens (ranked on the 387th position) and especially OF horses, which were positioned at the middle-left of the network, were less influential. In contrast, the population network associated with F_ROH_ and individual admixture levels of each horse at supervised clustering K = 5 revealed that OF horses are highly inbred, while in general admixed horses were less inbred (Fig. 3d). However, in this context, a highly inbred grey horse with an Arabian admixture proportion of 3% stood out.

### Comparison between pedigree-based and genomic derived inbreeding and admixture proportions of FM horses

**Fig. 4:**
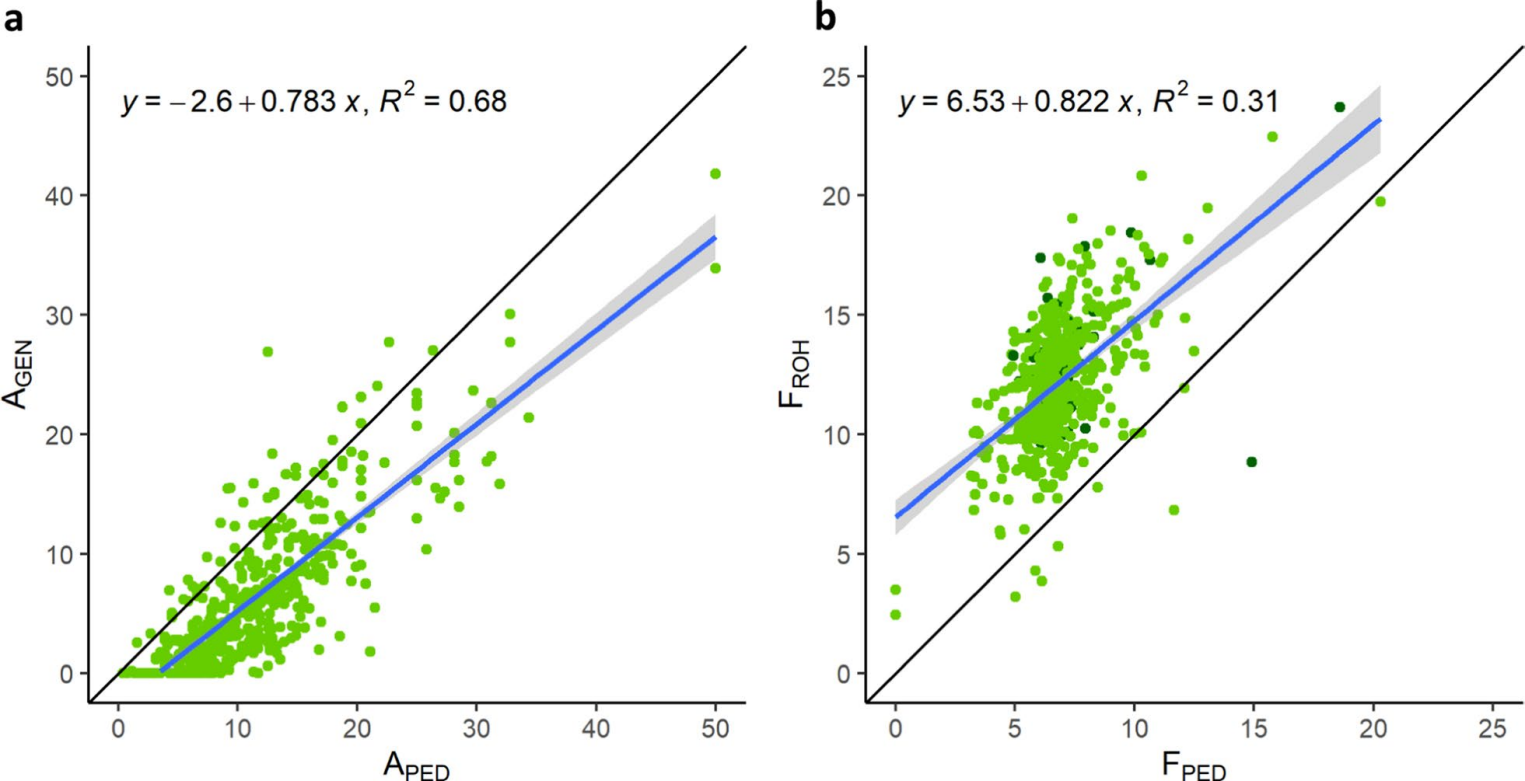
Comparison between genomic and pedigree-based estimates. **a** Linear regression of the relationship between the pedigree-based admixture (A_PED_) and the genomic admixture (A_GEN_) from the supervised model at K = 5; and **b** linear regression of the relationship between the pedigree-based inbreeding coefficient (F_PED_) and the genomic inbreeding coefficient F_ROH_. The OF horses are represented in dark green, the FM in lighter green

The correlation between genomic admixture levels derived from the unsupervised model (K = 2) and from the supervised model (K = 5) was 0.99. Supervised admixture levels (A_GEN_) were compared with pedigree-based admixture (A_PED_) in FM only, as OF was used as reference. The mean A_PED_ was 11.50 ± 6.59, with a minimum of 0.39 and a maximum of 50.00 for the two F1 crosses. The mean A_GEN_ was 6.40 ± 6.26, with a minimum of 0.004 and a maximum of 41.81, nearly half of the mean A_PED_. Despite this large discrepancy, the Pearson’s product-moment correlation between A_GEN_ and A_PED_ was 0.82 (R^2^ = 0.68, Fig. 4a) and highly significant (p < 0.0001). The intercept was − 2.60, meaning that A_GEN_ was generally only 2.60% lower than A_PED_, suggesting individuals with large divergences between the two values. Among the individuals with a higher A_GEN_ than A_PED_, horses with Don Fernando (F2 to Doktryner) ancestry stood out: the largest difference was seen in Don Fernando’s direct son (F3), with 15% greater A_GEN_ than A_PED_.

The mean pedigree-based inbreeding (F_PED_) was 6.84 ± 1.83 for OF and FM combined, while the mean F_ROH_ was 12.15 ± 2.68. The Pearson’s product-moment correlation between F_ROH_ and F_PED_ was 0.56 (R^2^ = 0.31) and highly significant (p < 0.0001). The intercept for the linear regression between F_ROH_ and F_PED_ was 6.53, meaning that F_ROH_ was generally 6.53% higher than F_PED_, i.e. nearly double (Fig. 4b). However, by adding the information of sire and dam to the regression equation (F_ROH_ ~ F_PED_ + sire + dam), R^2^ increased to 0.79.

### ROH patterns and distribution

The number of ROH segments was significantly different between all breeds, except between OF and WB (Table 1). Franches-Montagnes had the lowest mean number of ROH segments (N_ROH_), TB the highest. Mean total length of ROH segments (S_ROH_) significantly differed between all breeds, except between OF and SA. The shortest mean average length was for TB, the longest for OF. The mean L_ROH_ for FM and OF significantly differed from that of the other four breeds. The mean F_ROH_ differed between all breeds: TB had the highest mean F_ROH_, followed by AR, SA, OF, FM, and WB. Old-type FM had more ROH segments, but they were not significantly longer on average (L_ROH_) compared to FM. Therefore, OF had a longer S_ROH_ and were more inbred than FM.

**Table 1:** Summary statistics for number (N_ROH_), total length (S_ROH_), and average length (L_ROH_) of ROH segments, and of genomic inbreeding (F_ROH_)

Breed	n	Mean	SD	Median	Min	Max
TB	64					
N_ROH_		343.10^a^	58.47	351.50	64.00	432.00
S_ROH_ (Mb)		436.14^a^	111.98	458.98	44.17	602.94
L_ROH_ (Mb)		1.26^a^	0.25	1.26	0.69	1.95
F_ROH_ (%)		19.12^a^	4.91	20.12	1.94	26.43
AR	136					
N_ROH_		264.20^b^	20.39	264.00	210.00	313.00
S_ROH_ (Mb)		357.10^b^	54.48	347.60	204.00	503.10
L_ROH_ (Mb)		1.35^a^	0.16	1.33	0.97	1.84
F_ROH_ (%)		15.65^b^	2.39	15.24	8.94	22.06
SA	32					
N_ROH_		243.30^c^	25.64	248.50	172.00	287.00
S_ROH_ (Mb)		312.20^c^	74.62	308.10	157.60	462.30
L_ROH_ (Mb)		1.27^a^	0.21	1.23	0.90	1.69
F_ROH_ (%)		13.69^c^	3.27	13.51	6.91	20.27
WB	514					
N_ROH_		191.80^d^	27.02	190.00	54.00	287.00
S_ROH_ (Mb)		250.74^e^	54.58	247.66	37.47	445.88
L_ROH_ (Mb)		1.30^a^	0.19	1.30	0.69	2.33
F_ROH_ (%)		10.99^e^	2.39	10.86	1.64	19.55
OF	44					
N_ROH_		187.50^d^	22.94	182.00	158.00	246.00
S_ROH_ (Mb)		305.00^c^	62.73	297.90	201.70	540.40
L_ROH_ (Mb)		1.63^b^	0.27	1.63	1.04	2.31
F_ROH_ (%)		13.37^c^	2.75	13.06	8.85	23.69
FM	478					
N_ROH_		171.00^e^	25.81	170.00	69.00	318.00
S_ROH_ (Mb)		274.66^d^	60.50	272.52	55.59	512.15
L_ROH_ (Mb)		1.61^b^	0.29	1.61	0.81	2.84
F_ROH_ (%)		12.04^d^	2.65	11.95	2.44	22.45

AR: Purebred Arabian; SA: Shagya Arabian; TB: Thoroughbred; WB: European Warmblood; FM: Franches-Montagnes; OF: old-type Franches-Montagnes

^a−e^Means not sharing any letter were significantly different between breeds based on the Tukey-HSD test at the 5% level of significance

**Fig. 5:**
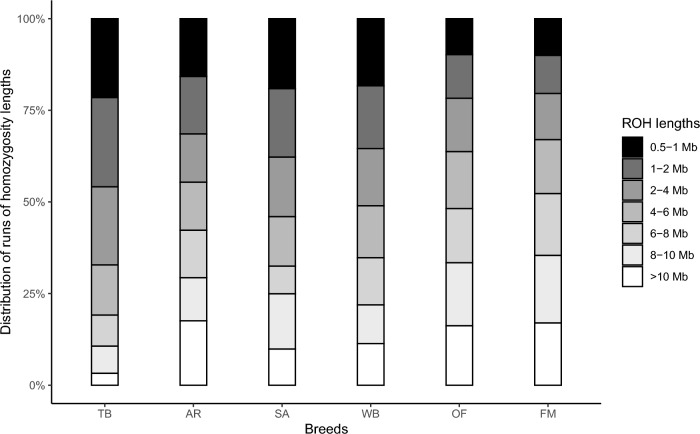
Distribution of the length of runs of homozygosity (ROH) segments. Length classes: 0.5–1, > 1–2, > 2–4, > 4–6, > 6–8, > 8–10 and > 10 Mb. Breeds: TB: Thoroughbred; AR: Purebred Arabian; SA: Shagya Arabian; WB: Warmblood; OF: old-type Franches-Montagnes; FM: Franches-Montagnes

While the total mean and total length of ROH segments was highest in TB, AR had highest proportion of ROH segments with a length > 10 MB, followed by FM and OF (Fig. 5). Warmblood and SA also had a high frequency of short ROH segments (0.5–2 MB), but a slightly higher proportion of ROH segments of intermediate length (4–8 MB) for WB and of long ROH segments (8–10 MB) for SA. Old-type FM had a majority of short to medium length ROH segments, while FM had proportionally more very long segments.

### ROH islands

There were no ROH islands on ECA13, ECA20, and ECA24 to ECA31. Thoroughbred had the most ROH islands (n = 78, Table 2), which concurs with results based on N_ROH_, i.e. breeds with more N_ROH_ also had more ROH islands. Warmblood had no ROH islands. Old-type FM had the longest ROH island (2.46 Mb). The chromosomal location and genes contained in the ROH islands were summarised by breed (see Additional file 2: Table S1, Additional file 3: Table S2, Additional file 4: Table S3, Additional file 5: Table S4, Additional file 6: Table S5). Not all loci in the ROH islands were annotated. The results from the GO analysis for each breed are summarized in Additional file 7: Table S6, Additional file 8: Table S7, Additional file 9: Table S8, Additional file 10: Table S9, and Additional file 11: Table S10. Most notably, genes involved in the biological process *epithelial cell differentiation (GO:0030855)* were overrepresented in AR (see Additional file 7: Table S6) and those in the biological process *embryonic skeletal system morphogenesis (GO:0048704)* in FM (see Additional file 11: Table S10) and OF (see Additional file 10: Table S9).

**Table 2:** Summary of runs of homozygosity (ROH) islands per breed

Breed	Total length of ROH islands (Mb)	Number of ROH islands	Number of genes in ROH islands (unmapped loci)	Number of private ROH islands
AR	16.42	26	186 (124)	9
SA	17.00	39	184 (104)	25
TB	33.05	78	314 (228)	70
OF	6.64	11	91 (39)	7
FM	4.89	7	106 (40)	0

European Warmblood did not have any ROH islands

AR: Purebred Arabian; SA: Shagya Arabian; TB: Thoroughbred; FM: Franches-Montagnes; OF: old-type Franches-Montagnes

There were 24 ROH islands that overlapped between at least two breeds (Table 3). One ROH island on ECA3 was shared between FM and Arabian breeds. Old-type FM shared ROH islands with FM, AR and SA and one with TB; FM shared islands with AR and SA, and the two Arabian breeds shared islands with TB.

**Table 3:** Overlaps of runs of homozygosity (ROH) islands (beginning and end in bp, length in Mb) between breeds, including annotated genes

Chr	Begin	End	Length	Breed sharing ROH island	Annotated genes within overlapping ROH island
1	30,971,234	31,479,608	0.51	FM, OF	*HPSE2, HPS1, PYROXD2, R3HCC1L, LOXL4*
2	27,876,021	28,404,281	0.53	FM, SA	*PTAFR, EYA3, XKR8, SMPDL3B, RPA2, THEMIS2, PPP1R8, STX12, FAM76A, IFI6, FGR, AHDC1*
3	120,431,916	120,437,951	0.01	OF, SA	–
3	120,437,951	120,536,237	0.10	FM, OF, SA	–
3	120,536,237	121,351,057	0.81	AR, FM, SA, OF	*NKX1-1, UVSSA, MAEA, CTBP1, SPON2, RNF212, FGFRL1, IDUA, SLC26A1, DGKQ, TMEM175, GAK, CPLX1, PCGF3, SLC49A3, MYL5, ATP5ME, PDE6B, PIGG*
4	52,661,137	52,750,288	0.09	OF, TB	–
4	55,776,953	55,819,015	0.04	AR, TB	–
7	41,107,447	41,434,416	0.33	AR, SA	*NTM*
7	41,434,416	41,938,373	0.50	AR, SA, TB	*NTM, OPCLM*
7	41,938,373	42,375,355	0.44	AR, TB	*OPCLM*
9	32,376,098	32,616,875	0.24	AR, SA	–
9	32,770,915	32,889,584	0.12	AR, SA	–
11	21,898,983	22,490,489	0.59	AR, SA	*KRT10B, KRT28, KRT27, KRT26, KRT25, KRT24, KRT222, SMARCE1, CCR7, TNS4, IGFBP4, TOP2A, GJD3, RARA, CDC6, WIPF2, RAPGEFL1, CASC3, MSL1, NR1D1, THRA*
11	23,273,876	25,588,903	2.32	FM, OF	*FBXO47, LASP1, RPL23, CWC25, PIP4K2B, PSMB3, PCGF2, CISD3, MLLT6, EPOP, SRCIN1, ARHGAP23, SOCS7, GPR179, MRPL45, NPEPPS, KPNB1, TBKBP1, TBX21, OSBPL7, MRPL10, LRRC46, SCRN2, SP6, SP2, PNPO, PRR15L, CDK5RAP3, COPZ2, NFE2L1, CBX1, SNX11, SKAP1, HOXB1, HOXB2, HOXB3, HOXB5, HOXB6, HOXB7, HOXB8, HOXB9, HOXB13, TTLL6, CALCOCO2, ATP5MC1, UBE2Z, SNF8, GIP, IGF2BP1, B4GALNT2, GNGT2, ABI3, PHOSPHO1, ZNF652, PHB, NGFR, NXPH3, SPOP*
11	26,192,181	26,426,827	0.23	AR, FM	*ACSF2, CHAD, RSAD1, MYCBPAP, EPN3, SPATA20, CACNA1G, ABCC3, ANKRD40, LUC7L3*
11	26,485,440	26,516,656	0.03	AR, FM	*WFIKKN2*
11	31,314,776	31,570,264	0.26	FM, SA	*NOG, DGKE*
11	31,361,576	32,109,154	0.75	AR, SA	*DGKE, TRIM25, COIL, SCPEP1, AKAP1, MSI2*
14	27,144,280	27,427,890	0.28	AR, TB	*PDGFRB, CSF1R, HMGXB3, TIGD6, SLC26A2, PDE6A, PPARGC1B*
14	27,439,297	27,500,357	0.06	AR, TB	*PPARGC1B*
18	49,358,178	49,443,663	0.09	AR, SA	*SSB, METTL5, UBR3*
18	49,443,663	49,527,351	0.08	AR, SA, TB	*UBR3*
18	49,527,351	49,917,777	0.39	AR, SA	*UBR3, MYO3B*
22	1,503,919	1,695,123	0.19	AR, TB	–

Chr: chromosome number; AR: Purebred Arabian; SA: Shagya Arabian; TB: Thoroughbred; WB: European Warmblood; FM: Franches-Montagnes; OF: old-type Franches-Montagnes

### Genotype frequencies of functional SNPs

**Fig. 6:**
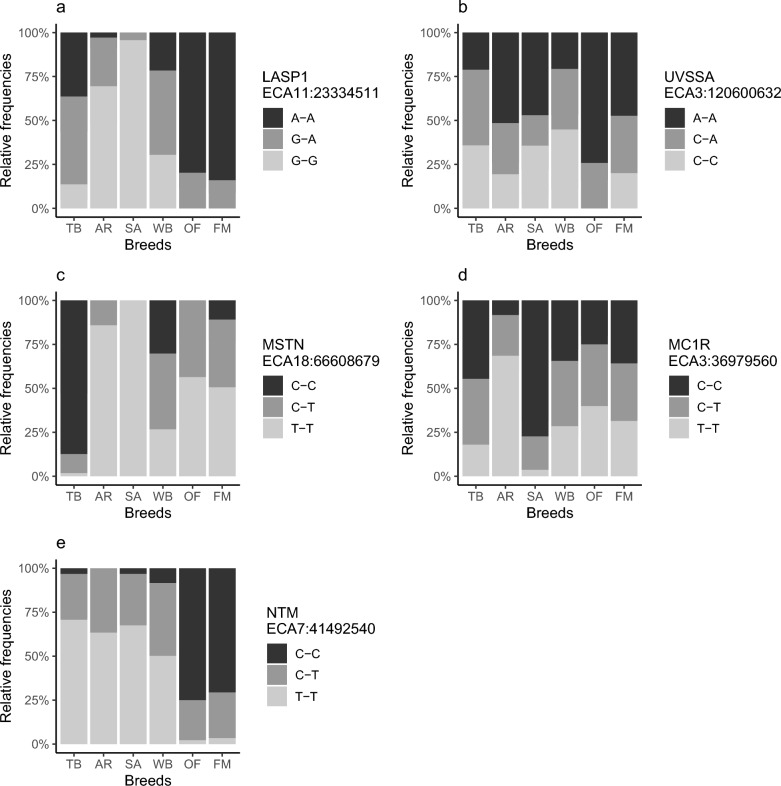
Frequencies of SNP genotypes with known functions in the six breeds  Thoroughbred (TB), Purebred Arabian (AR), Shagya Arabian (SA), European Warmblood (WB), Old-type Franches-Montagnes (OF) and Franches-Montagnes (FM). **a** The A allele of the *LASP1* SNP was associated with large sized horses; **b** the A allele from the *UVSSA* SNP was associated with higher UV exposure; **c** the C allele of the *MSTN* SNP was associated with better performance at shorter racing distance; **d** the T allele of the *MC1R* SNP was associated with the chestnut coat colour when homozygous; and **e** the C allele of the *NTM* SNP was associated with more race starts in Thoroughbred racehorses

We calculated the allele frequencies of functional SNPs in the following genes: *LASP1, UVSSA, MSTN, MC1R,* and *NTM* (Fig. 6). For *LASP1*, which has previously been associated with body size [15, 43], FM had the highest frequency of the “big” allele, even higher than OF, followed by TB and WB. Warmblood horses had the highest frequency of the *UVSSA* allele that was previously associated with lower UV exposure and therefore adaptation to Northern climates [14]. Old-type FM horses had the highest frequency of the allele associated with increased UV exposure, e.g. in Mountain ranges. The homozygous state for *MC1R*, expressing the chestnut coat colour, had the highest frequency AR, OF, FM, and WB. The majority of TB horses were homozygous for the *MSTN* allele, which has been shown to be beneficial for short distance races (C–C genotype) [44] while all SA and the majority of AR horses were homozygous for the opposite allele. For the intronic SNP for *NTM,* which was previously associated with the number of race course starts and therefore with precocity in TB [45], OF had the highest proportion of the favourable C allele, while TB had only few homozygous C–C horses. Franches-Montagnes horses with C–C genotypes had on average lower A_PED_ compared to C–T and T–T horses (see Additional file 12: Figure S2).

## Discussion

### High-resolution population structure of FM horses

Genetic forces (e.g. admixture and founder signatures) that have shaped the complex population structures of horses are currently inferred separately from each other using either parametric (e.g. Admixture) or non-parametric approaches (e.g. PCA). Recently, network-based cluster approaches, like NetView, are regaining favour for uncovering fine-scale population structures of horses [15, 17, 46]. The NetView approach can be used to visualize the relatedness of individuals without the need for any modelling of the genotype data [38]. Furthermore, it has been demonstrated that including individual admixture levels, genomic inbreeding, and genetic contributions in the network visualization describes a three-step procedure, which provides a high-resolution population structure without requiring prior ancestry information [35, 46].

Here, we applied the aforementioned three-step procedure to ascertain fine-scale population structure of FM horses. We showed that population substructures, as detected by model-based clustering, can be either associated with a different genetic origin or the progeny of most influential sires. For such complex population structures, we illustrated that identification of key contributors in combination with network visualization can shed light on the interpretation of admixture results, especially in situations where the cross-validation error estimation fails to provide an optimal number of clusters.

With regards to the formation of the FM breed, the high-resolution population structure analysis revealed that admixed FM horses showed unexpected high TB admixture levels, especially horses that descended from the Purebred Arabian stallion Doktryner. To clarify the origin of the DON line, further research is needed, including sequence analyses of the Y chromosome of male descendants [47–50]. Based on the identification of key contributors, we were also able to show that admixed FM horses (including one F1 sire from the N line and its progeny) had a strong impact on the current FM breeding population. Concerning the maintenance of genetic diversity in the breed, we further noticed that some FM individuals had lower F_ROH_ and fewer ROH segments (essentially the F1 and F2 crosses from the last introgressions), thereby decreasing the mean F_ROH_. However, the frequency of very long ROH segments was high in both breeds (longer than 8 MB for more than 25% of segments). Both OF and FM appeared to have been genetically bottlenecked. For FM, it was mainly due to the intensive selection of stallions and breeding of rare coat colours (e.g. grey). In OF, the limitations imposed by the pure-breeding efforts has considerably restricted the gene pool. To prevent these inbreeding activities, we suggest to integrate OF horses in the FM breeding population and to encourage breeders to target traits other than rare coat colour, as selection for rare coat colour has been proven to be detrimental to maintaining genetic diversity [15].

### Identification of ROH patterns and FROH in FM horses and other breeds

In our study, F_ROH_ for OF and FM was nearly twice as high than F_PED_ for the majority of the horses, and higher than expected based on previous studies on FM using 50 K data, in which the mean F_PED_ was slightly (0.2%) higher than F_ROH_ [51]. However, the medium correlation between F_ROH_ and F_PED_ (r < 0.60) was similar to that in other livestock species such as goats [52], sheep [9], and cattle [53, 54]. In general, the mean F_ROH_ of a population tends to be overestimated compared to F_PED_: higher F_ROH_ than F_PED_ have previously been reported in the Mangalarga Marchador [55], Italian Heavy Draught Horse [21], the Noriker, and to a much lesser extent in Haflinger horses [46]. Considering the depth of the pedigree data in the FM (> 20 generations), we suspect that both pedigree errors and selection of ROH settings affect the level of agreement between F_ROH_ and F_PED_. Currently, there are no standards for ROH settings in livestock [56] or horses. For the present study, we chose the same settings as previously applied in the study of other European horse breeds [14, 15, 17, 23]. We allowed for one heterozygous SNP per segment but phased the data to remove missing calls. However, as the genotype data is derived from SNP arrays, a further bias is introduced as the applied overlapping windows approach assumes that SNPs that are not included in the array are non-variants, which could cause an overestimation of ROH [56]. This will have little impact on the comparison of F_ROH_ between breeds in our study, but might affect the observed agreement (R^2^) between F_ROH_ and F_PED_ of FM horses. However, by including information on parentage, the R^2^ between F_ROH_ and F_PED_ increased considerably, as previously demonstrated [51]. Therefore, including parentage information in the estimation of F_ROH_ currently likely provides the most accurate information on inbreeding for conservation purposes.

With regards to other breeds, the TB had the most ROH segments, but they were also the shortest, indicative of historical inbreeding and consistent with an old established breed following a closed studbook [16, 57–60]. However, the cause for the higher F_ROH_ of TB compared to all other breeds may lie in the geographic origin of the samples, considering the strong bottleneck in the founder population of Australian TB [57]. The SA had the second shortest mean ROH segment lengths, and the third most ROH segments, indicative of a slightly less inbred population than TB. The AR had significantly more ROH segments, but they were not significantly longer than those of the SA population, although the AR had the highest proportion of very long ROH segments (> 10 Mb). The WB had ROH that were consistent with a breeding population with an open studbook, i.e. generally low inbreeding with some individual exceptions.

### Runs of homozygosity islands and gene functions

We found no ROH islands for WB, in contrast to previous studies [13, 19, 21], which may be because we regrouped two European WB subpopulations (Swiss and Swedish) and by the selection of ROH settings that differed from the previous studies. The TB had many ROHs and these contained over 300 annotated genes (see Additional file 4: Table S3), including genes that were previously associated with memory formation (*FSTL4*), bone formation, lung function and development (*WNT2B*), muscle cell proliferation and regulation of myogenesis (*KCNRG, MSTN*), and hip conformation (*IRX2*) [61]. The ROH islands contained several interleukin genes (*IL4, IL5, IL13*), which, together with their associated receptors, have been implicated in inflammatory processes such as recurrent airway obstruction and summer-pasture associated obstructive pulmonary disease (*IL4, IL13* [62–64]) or insect bite hypersensitivity (*IL5* [65–67]). There were also three Insulin-like growth factor binding protein genes (*IGFBP1, IGFBP3,* and *IGFBP6*), which are involved in somatic cell growth and muscle cell distribution, and were previously identified in breed-specific ROH islands including German and Swedish WB horses [13, 19] as well as French Trotters [14].

The ROH islands for the two Arabian breeds (AR and SA) were previously described by Grilz-Seger et al. [14] and will therefore be only briefly discussed, although there were slight differences in the length and position of these ROH islands compared to this previous study due to the remapping of the SNP positions using the new EquCab3.0 reference and to the specific subset in the AR population investigated here (only Doktryner descendants) (see Additional file 2: Table S1 and Additional file 3: Table S2). The presence of a large cluster of Keratin genes, which are associated with consistency of skin, hair, and structures such as the hoof capsule, remains notable in both Arabian breeds. The GO term *epithelial cell differentiation (GO:0030855)* was in fact enriched in AR*.* A recent study also identified *ZFMP1,* a gene within an AR-specific ROH island, as a candidate gene for hoof health in Mongolian horses [68]. Overall, the presence of multiple genes involved in hoof structure suggests that the identified ROH islands may contribute to the overall hoof health of AR. Some genes found in AR- or TB-specific ROH islands were associated with performance: *CBLB* is considered a negative regulator of the insulin response to exercise and was identified in a transcriptomic study of Arabian horses in training [69]. The gene *PPARGC1B* was present in islands for AR and TB and was previously associated with endurance in horses [70] and humans [71]. Unsurprisingly, SA and AR shared several ROH islands, considering that SA was classified as a variety of Arabian until 1978 [72].

The well-characterised *MSTN* gene was present in the ROH islands for TB. The high frequency of the C-allele for *MSTN* in TB is consistent with the popularity of shorter races that are common in Australasia, while the oriental breeds (SA and AR especially) are mostly used in endurance racing and had a high frequency of the T-allele. This SNP was also associated with the distribution of muscle fibre types (C alleles for type IIA and IIX, T alleles for type I) in TB, but not in Belgian Draught horses, due to differences in linkage disequilibrium [73]. Generally, TB have higher proportions of fast twitch type IIA muscle fibres that are advantageous for anaerobic activity over a distance of 1000 m, while AR have high amounts of type I muscles fibres, which are used for aerobic endurance work [74]. It was expected that FM would have more T alleles and therefore more type I muscle fibres, which are solicited in draught training [75], similar to other draught breed such as the Belgian draught horse [73]. However, this hypothesis needs to be confirmed by muscle biopsies, as the exact interplay between the *MSTN* gene and muscle fibre type distribution has not been completely elucidated [73], and muscle fibre type distribution has not yet been studied in FM.

The FM had only few ROH islands compared to AR, SA, and TB (see Additional file 5: Table S4). The long ROH island for OF and FM on ECA11 (2.32 Mb), which was not shared with the other breeds, contained two genes that were previously identified in ROH islands in Noriker horses *(LASP1)* and in Noriker, Posavina, Lipizzaner, and Gidran horses (*HOXB* cluster) [14], as well as in German Warmblood horses [19]. Homeobox genes such as the *HOXB* complex are critical for development of the spine and appendages during embryological development [76]. It is therefore not surprising that the GO term *embryonic skeletal system morphogenesis (GO:0048704)* was enriched in OF and FM, while *embryonic skeletal system development (GO:0048706)* and *skeletal system morphogenesis (GO:0048705)* were additionally enriched in FM. Genes from the *HOXB* cluster were already under selection pressure in Europe during the Sassanid Persian expansion between the 7th to 9th century [77]. The FM, Noriker, Posavina, and Lipizzaner horses tend to favour a short back in relation to limb length, which might be the reason that  the *HOXB* gene cluster became relatively fixed in these breeds. However, without a definitive phenotype-genotype association, this remains speculative. The *NOG* gene, found in an ROH island for SA and FM but not in OF, is also associated with the length of phalanges [78, 79], which would suggest that breeders are selecting against shorter legs in FM compared to OF. The *LASP1* gene has previously been identified as one of the major genes affecting size of the horse, particularly wither’s height [43]. However, based on the allele frequencies of the SNP that was previously identified by Makvandi-Nejad et al. [43], we concur with Grilz-Seger et al. [15] that the *LASP1* gene is probably involved in body size (heavy or light type) rather than height itself. The FM had the highest frequency of “big” alleles, despite height at withers being limited to 160 cm in the breeding standards. Meanwhile, WB horses, who at least in Switzerland have to exceed 160 cm at the withers to be admitted to the studbook, have a low frequency of “big” alleles. However, SA had the lowest frequency of “big” alleles, although the SA should have a larger frame than the AR, and the “big” allele is even more frequent in FM than in OF [72]. This discrepancy is likely explained by other variants that did not appear in the ROH islands, such as *ZFAT* [15].

In an OF-specific ROH island (see Additional file 6 Table S5), we identified the gene Serotonin receptor 1B *(HTR1B* or *5-HT1B)* on ECA10. Serotonin is an important neurotransmitter associated with coping styles in humans and animals [80]. *HTR1B-*knockout mice have been shown to be more reactive and less anxious than the wild-type mice [81] and more reactive to rewards [82]. Knockout rats are less likely to be startled but will react more forcefully under prolonged stress, indicative of a reactive coping style [83]. In horses, more reactive horses show no obvious reactions to aversive stimuli, which corresponds to the animals being known as “cold-blooded” [84, 85]. It is possible that the *HTR1B* gene has been under selection in OF as a working draught horse, for which strong flight-or-fight responses are undesirable. As this ROH island was not shared with FM, this would also imply that introgressions and selection towards a lighter type of horse influenced the genetic makeup of the breed as well as behaviour, towards a more proactive horse. However, *HTR1B*-knockout mice were also associated with an increase in aggressive behaviour [86], so it is not clear how the homozygous haplotype of *HTR1B* in the OF affects their behaviour. Considering the complexity of behaviour, more studies are needed to better interpret these results.

We identified another ROH island that has been associated with behaviour: SA, AR, and TB shared an ROH island containing the genes *NTM* and *OPCLM*, which belong to the IgLON protein family that regulate development of neuronal projections [87]. While downregulation of *OPCLM* has been associated with schizophrenia in Han Chinese [88], certain *NTM* polymorphisms in humans were associated with IQ [89] or aggressiveness in childhood ADHD [90]. *NTM* knockout mice were less fearful, moved more and reacted less to averse stimuli [91, 92], which might be favourable traits in sport horses, while *OPCML* knockout mice were less curious, had less spatial recognition and recognition memory in general, and showed less interest in their conspecifics [88]. In horses, the *NTM* gene has been one of the key genes involved in domestication [93] and has been found in selection signatures for racehorses such as TB [94] and Purebred Arabian horses [14]. Furthermore, this region on ECA7 has also been divergently selected between draught and light type breeds [95]. To better understand the direction of selection in *NTM*, we selected a SNP on the 670 K array that was previously described by McGivney et al. [45] to be associated with the number of race starts in Thoroughbreds. It was suggested that the favourable allele was indicative of a calmer temperament in the horse, with these horses racing earlier in life and therefore more often [45]. In our study, the favourable C allele was overrepresented in OF and FM, and nearly absent in all other breeds, even TB. This concurs with the breeding goal for a horse with good character. However, it could also be shown (see Additional file 12 Figure S2) that the unfavourable allele is associated with higher A_PED_, suggesting that selection towards a lighter sport horse may affect the calm character the FM breed is known for.

## Conclusions

The FM is the last native Swiss breed and is clearly distinguishable from other historically introgressed breeds, whereas admixed FM horses had a strong impact on the current breeding population. The FM was less inbred than other purebred horse breeds, but suffered bottlenecks due to intensive selection of stallions, restrictive mating choices based on arbitrary definitions of pure breeding, and selection for rare coat colours. Genes embedded in ROH islands had known functions involved in conformation and behaviour, two traits highly valued by breeders. The unexpected high TB admixture proportions in FM need further investigation.

### Supplementary Information

Additional file 1: Figure S1. Identification of the optimal number of clusters (K). Illustration of the cross-validation error increasing K from 1 to 20.

Additional file 2: Table S1. Runs of homozygosity segments shared by more than 50% of Purebred Arabians. Table S1 presents the runs of homozygosity segments shared by more than 50% of Purebred Arabians including the length and position along the chromosomes. The annotated genes within the segments are also reported.

Additional file 3: Table S2. Runs of homozygosity segments shared by more than 50% of Shagya Arabians. Table S2 presents the runs of homozygosity segments shared by more than 50% of Shagya Arabians including the length and position along the chromosomes. The annotated genes within the segments are also reported.

Additional file 4: Table S3. Runs of homozygosity segments shared by more than 50% of Thoroughbreds. Table S3 presents the runs of homozygosity segments shared by more than 50% of Thoroughbreds including the length and position along the chromosomes. The annotated genes within the segments are also reported.

Additional file 5: Table S4. Runs of homozygosity segments shared by more than 50% of Old-type Franches-Montagnes. Table S4 presents the runs of homozygosity segments shared by more than 50% of Old-type Franches-Montagnes including the length and position along the chromosomes. The annotated genes within the segments are also reported.

Additional file 6: Table S5. Runs of homozygosity segments shared by more than 50% of Franches-Montagnes. Table S5 presents the runs of homozygosity segments shared by more than 50% of Franches-Montagnes including the length and position along the chromosomes. The annotated genes within the segments are also reported.

Additional file 7: Table S6. Gene ontology analysis for the Purebred Arabians. Table S6 presents the results from the gene ontology analysis for the genes present in the runs of homozygosity islands for the Purebred Arabians.

Additional file 8: Table S7. Gene ontology analysis for the Shagya Arabians. Table S7 presents the results from the gene ontology analysis for the genes present in the runs of homozygosity islands for the Shagya Arabians.

Additional file 9: Table S8. Gene ontology analysis for the Thoroughbred. Table S8 presents the results from the gene ontology analysis for the genes present in the runs of homozygosity islands for the Thoroughbred.

Additional file 10: Table S9. Gene ontology analysis for the old-type Franches-Montagnes. Table S9 presents the results from the gene ontology analysis for the genes present in the runs of homozygosity islands for the old-type Franches-Montagnes.

Additional file 11: Table S10. Gene ontology analysis for the Franches-Montagnes. Table 10 presents the results from the gene ontology analysis for the genes present in the runs of homozygosity islands for the Franches-Montagnes.

Additional file 12: Figure S2. Association between Percent of pedigree-based admixture proportion and *NTM* SNP genotype. The C allele of the *NTM* SNP was associated with more race starts in Thoroughbred racehorses. Boxplot representing the pedigree-based admixture based on the *NTM* SNP genotype.

## Data Availability

Anonymised data are available for academic purposes on signing a material transfer agreement.
