# Supplementary figures and images for: Using high-density SNP data to unravel the origin of the Franches-Montagnes horse breed
Source: Genet Sel Evol. 2024 Jul 10;56:53. doi: 10.1186/s12711-024-00922-6 (PMC11238448; doi:10.1186/s12711-024-00922-6)

## Admixture

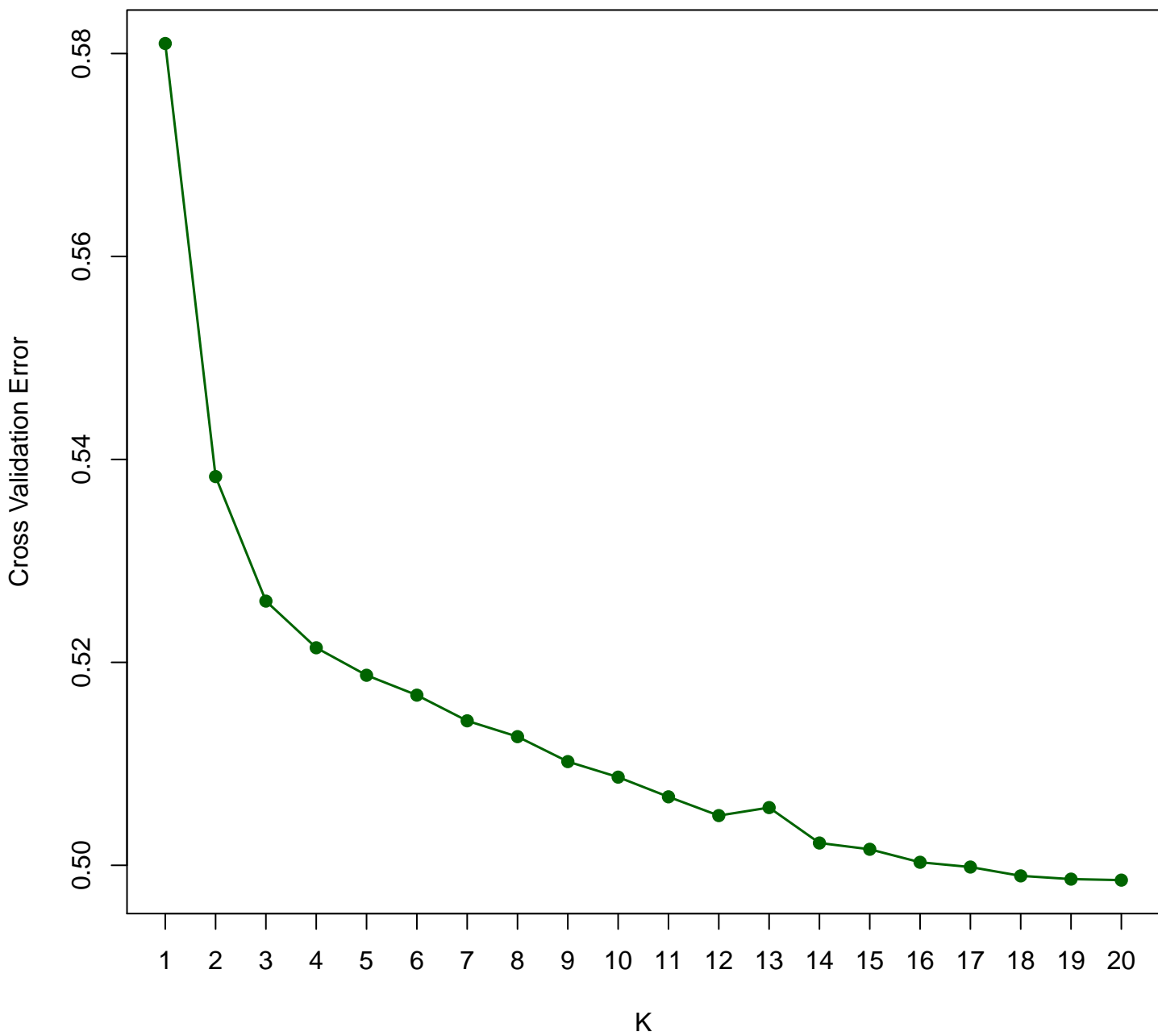

Supplement: Supplementary file 1 — Additional file 1: Figure S1. Identification of the optimal number of clusters (K). Illustration of the cross-validation error increasing K from 1 to 20. [file 12711_2024_922_MOESM1_ESM.pdf]

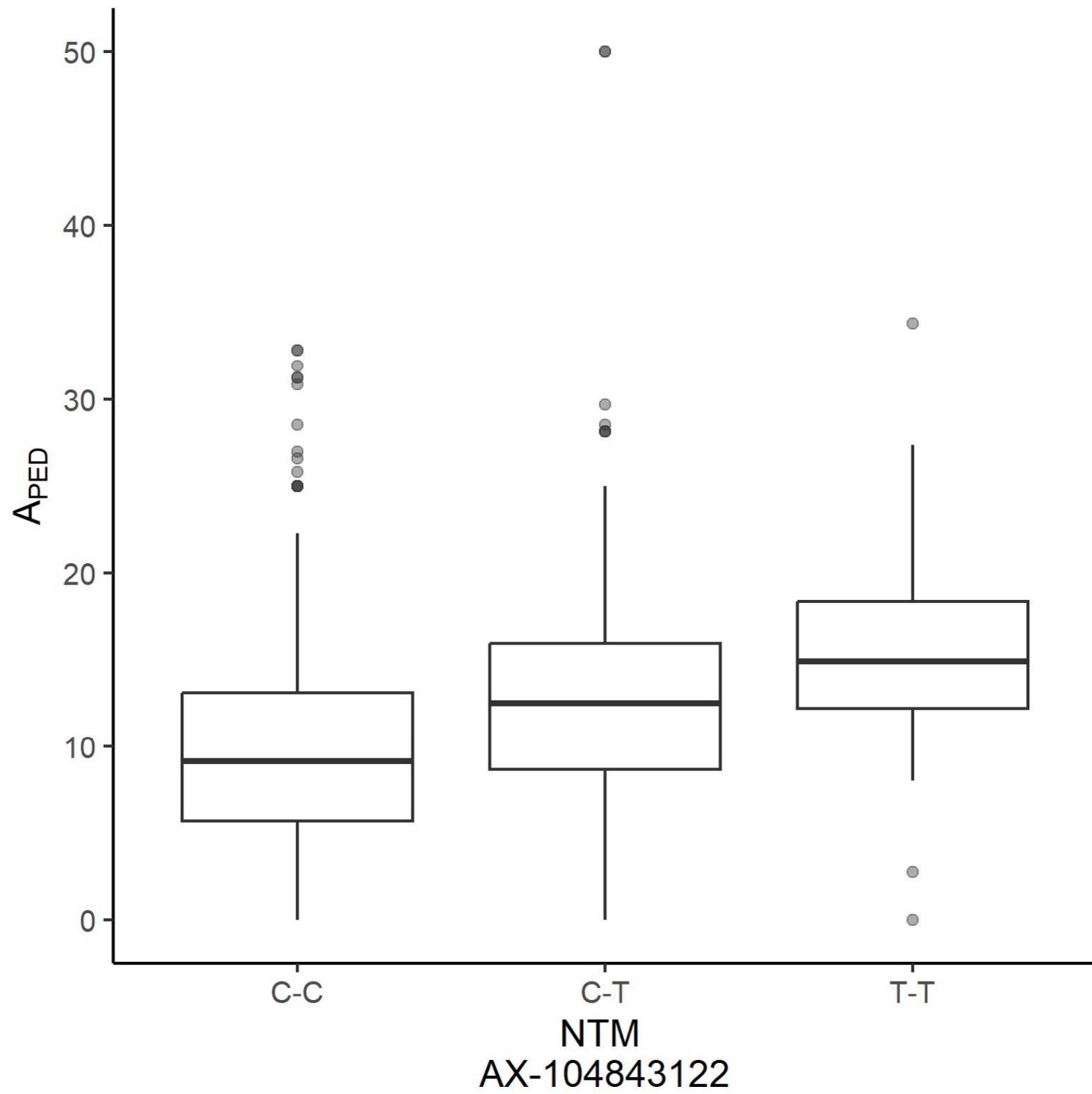

Supplement: Supplementary file 12 — Additional file 12: Figure S2. Association between Percent of pedigree-based admixture proportion and NTM SNP genotype. The C allele of the NTM SNP was associated with more race starts in Thoroughbred racehorses. Boxplot representing the pedigree-based admixture based on the NTM SNP genotype. [file 12711_2024_922_MOESM12_ESM.pdf]
