# Supplementary material for: Using high-density SNP data to unravel the origin of the Franches-Montagnes horse breed
Source: Genet Sel Evol. 2024 Jul 10;56:53. doi: 10.1186/s12711-024-00922-6 (PMC11238448; doi:10.1186/s12711-024-00922-6)
Supplement: Supplementary file 2 — Additional file 2: Table S1. Runs of homozygosity segments shared by more than 50% of Purebred Arabians. Table S1 presents the runs of homozygosity segments shared by more than 50% of Purebred Arabians including the length and position along the chromosomes. The annotated genes within the segments are also reported. [file 12711_2024_922_MOESM2_ESM.pdf]

Table S1: Runs of homozygosity segments shared for over 50% of Purebred Arabians.

Chr.	Length (MB)	Begin	End	Annotated genes
2	1.14	100852761	101993852	*PGRMC2, LARP1B, ABHD18, MFSD8, PLK4, HSPA4L, SLC25A31, INTU*
3	0.55	35410588	35959266	*ZNF469, ZFPM1, ZC3H18, IL17C, CYBA, MVD, SNAI3, RNF166, CTU2, PIEZO1*
3	1.93	36856010	38783069	*FANCA, SPIRE2, TCF25, MC1R, TUBB3, DEF8, CENPBD1, DBNDD1, GAS8, PRDM7, CENPE, BDH2, SLC9B2, SLC9B1, CISD2, UBE2D3, MANBA, NFKB1, SLC39A8, BANK1*
3	0.81	120536237	121351057	*NKX1-1, UVSSA, MAEA, CTBP1, SPON2, RNF212, FGFRL1, IDUA, SLC26A1, DGKQ, TMEM175, GAK, MIR9070, CPLX1, PCGF3, SLC49A3, MYL5, ATP5ME, PDE6B, PIGG*
4	0.87	55111503	55984784	*IGF2BP3, TRA2A, CCDC126, FAM221A, STK31, NPY*
5	0.78	48918960	49698979	*IGSF3, CD58, ATP1A1, MAB21L3, SLC22A15, NHLH2, CASQ2*
7	1.27	41107447	42375355	*NTM, OPCML*
7	0.17	52293923	52464624	-
7	0.04	52805181	52849963	-
7	0.09	53235458	53321837	-
7	0.03	53368641	53397997	-
7	0.53	53441389	53972641	-
9	0.38	32376098	32759901	*PXDNL*
9	0.12	32770915	32889584	-
9	0.57	45650223	46219316	*LAPTM4B, MATN2, RPL30, ERICH5, RIDA, POP1, NIPAL2, KCNS2, STK3*
11	0.72	21768214	22490489	*KRT39, KRT23, KRT20, KRT12, KRT10A, KRT10B, KRT28, KRT27, KRT26, KRT25, KRT24, KRT222, SMARCE1, CCR7, TNS4, IGFBP4, TOP2A, GJD3, RARA, CDC6, WIPF2, RAPGEFL1, CASC3, MSL1, NR1D1, THRA*
11	0.98	26192181	27169261	*ACSF2, CHAD, RSAD1, MYCBPAP, EPN3, SPATA20, CACNA1G, ABCC3, ANKRD40, LUC7L3, WFIKKN2, TOB1, SPAG9, NME1, NME2, MBTD1, UTP18, CA10*
11	1.07	27917117	28986458	-
11	0.75	31361576	32109154	*DGKE, TRIM25, COIL, SCPEP1, AKAP1, MSI2*
14	0.28	27144280	27427890	*PDGFRB, CSF1R, HMGXB3, TIGD6, SLC26A2, PDE6A, PPARGC1B*
14	0.43	27439297	27866083	*PPARGC1B, ARHGEF37, CSNK1A1, IL17B, PCYOX1L, GRPEL2, AFAP1L1*
18	0.98	10299223	11275554	*INHBB, RALB, TMEM185B, EPB41L5, PTPN4*
18	0.68	49358178	50034758	*SSB, METTL5, UBR3, MYO3B*
19	0.45	53200565	53654053	*CBLB, ALCAM*
22	0.19	1503919	1695123	-
22	0.60	23865659	24470011	*KIF3B, ASXL1, NOL4L, COMMD7, DNMT3B, MAPRE1, EFCAB8, SUN5, BPIFB2, BPIFB6, BPIFB3, BPIFB4*
