# Supplementary material for: Using high-density SNP data to unravel the origin of the Franches-Montagnes horse breed
Source: Genet Sel Evol. 2024 Jul 10;56:53. doi: 10.1186/s12711-024-00922-6 (PMC11238448; doi:10.1186/s12711-024-00922-6)
Supplement: Supplementary file 3 — Additional file 3: Table S2. Runs of homozygosity segments shared by more than 50% of Shagya Arabians. Table S2 presents the runs of homozygosity segments shared by more than 50% of Shagya Arabians including the length and position along the chromosomes. The annotated genes within the segments are also reported. [file 12711_2024_922_MOESM3_ESM.pdf]

Table S2: Runs of homozygosity segments shared by over 50% of Shagya Arabians.

Chr.	Length (MB)	Begin	End	Annotated genes
1	0.02	9501779	9519364	-
1	0.64	107739971	108378967	*TARSL2, TJP1, FAM189A1*
2	1.06	27344713	28404281	*OPRD1, YTHDF2, GMEB1, TAF12, RAB42, RCC1, PHACTR4, MED18, SESN2, ATP5IF1, DNAJC8, PTAFR, EYA3, XKR8, SMPDL3B, RPA2, THEMIS2, PPP1R8, STX12, FAM76A, IFI6, FGR, AHDC1*
3	0.93	120425335	121351057	*NKX1-1, UVSSA, MAEA, CTBP1, SPON2, RNF212, FGFRL1, IDUA, SLC26A1, DGKQ, TMEM175, GAK, MIR9070, CPLX1, PCGF3, SLC49A3, MYL5, ATP5ME, PDE6B, PIGG*
6	0.18	8018928	8201303	*ZNF142, BCS1L, RNF25, STK36, TTLL4, CYP27A1, PRKAG3*
7	0.25	40755980	41002946	*NTM*
7	0.88	41060288	41938373	*NTM, OPCML*
8	0.60	24634587	25231545	*RHOF, SETD1B, HPD, PSMD9, WDR66, BCL7A, MLXIP, IL31, LRRC43, B3GNT4, DIABLO, VPS33A, CLIP1, ZCCHC8, RSRC2, KNTC1*
8	0.27	25825349	26097405	*KMT5A, RILPL2, SNRNP35, RILPL1, TMED2, DDX55, EIF2B1, GTF2H3, TCTN2, ATP6V0A2, DNAH10*
8	0.00	61839365	61839365	-
9	0.53	31284516	31817479	*RB1CC1, ALKAL1*
9	0.76	31853320	32616875	*ST18, PCMTD1*
9	0.22	32677402	32897046	*PXDNL*
9	0.52	33532618	34053277	*SNTG1*
9	0.87	40515338	41382074	*TRIQK*
9	0.58	41416774	41992448	*FAM92A, RBM12B, TMEM67*
9	0.76	41998238	42758961	*TMEM67, PDP1, CDH17, GEM, RAD54B, VIRMA*
9	0.03	42822721	42852068	*ESRP1, DPY19L4*
9	0.01	45565108	45576777	*MTDH*
9	0.92	45613807	46532261	*LAPTM4B, MATN2, RPL30, ERICH5, RIDA, POP1, NIPAL2, KCNS2, STK3*
9	0.61	79742728	80350559	*FAM135B, COL22A1*
11	0.72	21898983	22614034	*KRT10B, KRT28, KRT27, KRT26, KRT25, KRT24, KRT222, SMARCE1, CCR7, TNS4, IGFBP4, TOP2A, GJD3, RARA, CDC6, WIPF2, RAPGEFL1, CASC3, MSL1, NR1D1, THRA, MED24, CSF3, PSMD3, GSDMA, LRRC3C, ORMDL3, GSDMB*
11	0.00	22654340	22654340	*IKZF3*
11	0.05	22703722	22750090	*IKZF3, GRB7*
11	0.31	22880941	23186362	*CDK12, MED1, FBXL20, STAC2, RPL19, CACNB1, ARL5C, PLXDC1*
11	1.12	31314776	32434775	*NOG, DGKE, TRIM25, COIL, SCPEP1, AKAP1, MSI2, CCDC182*
11	0.04	32617562	32658713	*DYNLL2*
11	0.58	32803157	33383463	*LPO, MPO, TSPOAP1, RNF43, SUPT4H1, HSF5, MTMR4, TEX14, RAD51C, PPM1E*
11	0.23	34276336	34508997	*MED13*
11	0.01	34577854	34586693	*BRIP1*
11	0.12	34683002	34804064	*BRIP1*
15	1.04	80216896	81259084	*NBAS, FAM84A*
16	0.16	33620538	33782347	*ERC2*
16	0.35	41847777	42194451	*LTF, CCRL2, CCR5, CCR2, CCR3*
18	0.56	49358178	49917777	*SSB, METTL5, UBR3, MYO3B*
18	0.51	50413659	50924378	*TLK1, METTL8, DCAF17*
18	0.09	81688757	81776604	*CREB1, METTL21A*
19	0.07	34349123	34421059	*XXYLT1*
23	0.42	25249949	25673636	*GLIS3, SLC1A1, SPATA6L, CDC37L1, AK3, RCL1*
