# Supplementary material for: Using high-density SNP data to unravel the origin of the Franches-Montagnes horse breed
Source: Genet Sel Evol. 2024 Jul 10;56:53. doi: 10.1186/s12711-024-00922-6 (PMC11238448; doi:10.1186/s12711-024-00922-6)
Supplement: Supplementary file 4 — Additional file 4: Table S3. Runs of homozygosity segments shared by more than 50% of Thoroughbreds. Table S3 presents the runs of homozygosity segments shared by more than 50% of Thoroughbreds including the length and position along the chromosomes. The annotated genes within the segments are also reported. [file 12711_2024_922_MOESM4_ESM.pdf]

Table S3 : Runs of homozygosity segments shared by over 50% of Thoroughbreds.

Chr.	Length (MB)	Begin	End	Annotated genes
1	0.59	841540	1435208	*CFAP46, NKX6-2, INPP5A, PWWP2B*
1	1.14	22010452	23149101	-
1	0.17	33184195	33356486	*ZNF518A, CCNJ, CC2D2B*
1	0.02	33371412	33394390	*CC2D2B*
1	0.38	33415244	33792526	*ENTPD1, TCTN3, ALDH18A1, SORBS1*
1	0.37	41105674	41472996	*PAPSS2, MINPP1, SGMS1*
1	0.43	46911137	47336515	-
1	0.03	47375634	47405427	-
1	0.39	47421744	47812471	-
1	0.22	68536819	68761749	-
1	0.23	68768009	68999855	*URB2, TAF5L, ABCB10, NUP133, ACTA1*
1	0.45	71888373	72334036	*BMS1, ZNF248*
1	0.68	85013819	85690537	*GRID1*
1	0.64	93639301	94280015	*CRTC3, BLM, FURIN, FES, MAN2A2, HDDC3, UNC45A, RCCD1, PRC1, VPS33B, NGRN, GDPGP1, CIB1, SEMA4B, IDH2, ZNF710*
1	0.37	112717075	113088084	*GABRB3*
1	0.17	113530630	113703332	*GABRG3*
1	0.10	113767681	113866140	*GABRG3*
1	0.05	113952712	114006066	-
1	0.00	123226504	123229881	-
2	0.41	64662562	65067588	-
2	0.10	65086799	65187559	-
3	0.36	22526491	22886805	*DHX38, TXNL4B, DHODH, PKD1L3, IST1, ZNF821, ATXN1L, AP1G1, PHLPP2*
3	0.63	23444991	24072266	*HYDIN, VAC14, MTSS1L, IL34, SF3B3, COG4, FUK, ST3GAL2, AARS, EXOSC6, CLEC18B*
3	0.55	40028601	40581467	*DNAJB14, LAMTOR3, DAPP1, MTTP, TRMT10A*
3	0.13	58442558	58572539	-
4	0.78	15528412	16305907	*CCM2, NACAD, TBRG4, RAMP3, ADCY1, IGFBP1, IGFBP3*
4	0.49	44067176	44558669	*NDUFA4, PHF14*
4	0.01	44578375	44592736	-
4	0.99	52210255	53198512	*MACC1, ITGB8, ABCB5, SP8*
4	0.04	55776953	55819015	-
5	0.81	52075884	52883274	*SLC16A1, FAM19A3, PPM1J, RHOC, MOV10, CAPZA1, ST7L, WNT2B, CTTNBP2NL,*
5	0.15	54254470	54401315	*KCNA2, KCNA10, PROK1*
5	0.01	54467121	54473450	-
5	0.60	54607188	55203319	*SLC6A17, UBL4B, ALX3, STRIP1, AHCYL1, CSF1, EPS8L3, GSTM3, AMPD2, GNAT2, MIR197, GNAI3, GPR61, AMIGO1, ATXN7L2*
6	1.13	21644998	22775700	*AGAP1, GBX2, ASB18, IQCA1, ACKR3*
6	0.84	23669396	24511131	*LRRFIP1, RBM44, RAMP1, UBE2F, SCLY, ESPNL, KLHL30, ERFE, ILKAP, HES6, PER2, TRAF3IP1*
6	0.09	42866900	42960667	-
6	0.00	42965882	42966523	-
6	0.40	43084381	43480499	*ATF7IP, PLBD1, GUCY2C, WBP11, SMCO3, ART4*
6	0.52	68244053	68766546	*FMNL3, TMBIM6, NCKAP5L, BCDIN3D, FAIM2, AQP2, AQP5, AQP6, RACGAP1, ASIC1, SMARCD1, GPD1, CERS5, LIMA1*
6	0.61	70863528	71473496	*KRT8, KRT18, EIF4B, TNS2, SPRYD3, IGFBP6, SOAT2, CSAD, ZNF740, ITGB7, RARG, MFSD5, ESPL1, PFDN5, AAAS, SP7, SP1, AMHR2, PRR13, PCBP2, MAP3K12, TARBP2, NPFF, ATF7, ATP5MC2*
6	0.00	71855266	71855914	-
7	0.17	40386493	40560613	-
7	1.56	41434416	42997806	*NTM, OPCML*
7	1.02	43046689	44065860	*SPATA19, IGSF9B, JAM3, NCAPD3, VPS26B, ACAD8, THYN1, B3GAT1*
7	0.07	44109054	44181928	-
7	0.57	44418390	44988705	-

7	0.45	45886267	46334427	*ADGRE5, ADGRL1, PRKACA, SAMD1, MISP3, PALM3, IL27RA, RLN3, MIR1271B, RFX1, DCAF15, PODNL1, CC2D1A, NANOS3, ZSWIM4, MRI1, CCDC130*
7	0.96	46961963	47917776	*MAN2B1*
8	0.65	44239408	44892092	*ENOSF1, YES1, ADCYAP1*
9	0.44	67863639	68304060	*ATAD2, WDYHV1, FBXO32, KLHL38, ANXA13, FAM91A1, FER1L6*
9	0.02	70089528	70111792	-
10	0.01	15133223	15146131	-
11	0.50	2407702	2910649	*RPTOR, NPTX1, ENDOV, RNF213, SLC26A11, SGSH, CARD14, EIF4A3, GAA, CCDC40, TBC1D16*
11	0.05	23186362	23238557	*PLXDC1*
12	0.49	36456898	36942815	*MOB2, DUSP8, RNH1, PTDSS2, ANO9, SIGIRR, PKP3, B4GALNT4, IFITM5, PGGHG, NLRP6, PSMD13*
14	0.42	15982846	16398029	*MAT2B, HMMR, NUDCD2, CCNG1*
14	0.03	16437615	16467602	-
14	0.36	27144280	27500357	*PDGFRB, CSF1R, HMGXB3, TIGD6, SLC26A2, PDE6A, PPARGC1B*
14	0.99	40943485	41931301	*VDAC1, FSTL4, HSPA4, ZCCHC10*
14	0.49	41989512	42478605	*AFF4, LEAP2, GDF9, SHROOM1, SOWAHA, SEPT8, KIF3A, IL4, IL13, RAD50, IL5, IRF1, SLC22A5*
14	0.51	46642345	47156636	*MEGF10, MARCH3, LMNB1, TEX43, PHAX, ALDH7A1*
16	0.38	34619130	34998423	*CACNA2D3*
16	0.27	35029621	35299636	*CACNA2D3*
16	0.02	35321893	35341801	-
16	0.92	35390401	36312167	*SELENOK, ACTR8, IL17RB, CHDH, CACNA1D, DCP1A, TKT, PRKCD, RFT1, SFMBT1*
17	2.04	20626515	22665932	*KCNRG, TRIM13, SPRYD7, KPNA3, EBPL, ARL11, RCBTB1, SETDB2, CAB39L, CDADC1, MLNR, FNDC3A, CYSLTR2, RCBTB2, RB1, LPAR6, ITM2B*
18	0.06	30087303	30146071	-
18	0.18	42472474	42650850	*DPP4, GCG, FAP*
18	0.08	49443663	49527351	*UBR3*
18	0.82	49579653	50401745	*UBR3, MYO3B, SP5, ERICH2, GAD1, GORASP2, TLK1*
18	0.44	66605255	67042719	*MSTN, HIBCH, INPP1, MFSD6, NEMP2*
21	0.02	55559098	55579756	-
21	0.16	55620187	55776417	-
21	0.04	55810398	55845927	-
21	0.45	56708164	57162831	*IRX2*
22	0.56	1154739	1712638	*SYNDIG1*
23	0.77	33553984	34327444	*NFIB, ZDHHC21*
