# Supplementary material for: Using high-density SNP data to unravel the origin of the Franches-Montagnes horse breed
Source: Genet Sel Evol. 2024 Jul 10;56:53. doi: 10.1186/s12711-024-00922-6 (PMC11238448; doi:10.1186/s12711-024-00922-6)
Supplement: Supplementary file 5 — Additional file 5: Table S4. Runs of homozygosity segments shared by more than 50% of Old-type Franches-Montagnes. Table S4 presents the runs of homozygosity segments shared by more than 50% of Old-type Franches-Montagnes including the length and position along the chromosomes. The annotated genes within the segments are also reported. [file 12711_2024_922_MOESM5_ESM.pdf]

Table S4 : Runs of homozygosity segments shared by over 50% of Old-type Franches-Montagnes.

Chr.	Length (Mb)	Begin	End	Annotated genes
1	0.58	30925921	31506917	*HPSE2, HPS1, PYROXD2, R3HCC1L, LOXL4*
3	0.92	120431916	121351057	*NKX1-1, UVSSA, MAEA, CTBP1, SPON2, RNF212, FGFRL1, IDUA, SLC26A1, DGKQ, TMEM175, GAK, MIR9070, CPLX1, PCGF3, SLC49A3, MYL5, ATP5ME, PDE6B, PIGG*
4	0.09	52661137	52750288	-
8	0.50	38208512	38709040	*APCDD1, NAPG, PIEZO2*
10	0.16	31082208	31239467	*MYO6, IMPG1*
10	0.20	31314431	31514864	-
10	0.51	31996052	32503989	*HTR1B*
11	2.46	23273876	25734280	*FBXO47, LASP1, RPL23, CWC25, PIP4K2B, PSMB3, PCGF2, CISD3, MLLT6, EPOP, SRCIN1, ARHGAP23, SOCS7, GPR179, MRPL45, NPEPPS, KPNB1, TBKBP1, TBX21, OSBPL7, MRPL10, LRRC46, SCRN2, SP6, SP2, PNPO, PRR15L, CDK5RAP3, COPZ2, NFE2L1, CBX1, SNX11, SKAP1, HOXB1, HOXB2, HOXB3, HOXB5, HOXB6, HOXB7, HOXB8, HOXB9, HOXB13, TTLL6, CALCOCO2, ATP5MC1, UBE2Z, SNF8, GIP, IGF2BP1, B4GALNT2, GNGT2, ABI3, PHOSPHO1, ZNF652, PHB, NGFR, NXPH3, SPOP, SLC35B1, FAM117A, KAT7, TAC4*
11	0.07	30922916	30989489	-
15	0.57	9495555	10066795	*AFF3*
15	0.59	24560550	25147192	*CTNNA2*
