# Supplementary material for: Using high-density SNP data to unravel the origin of the Franches-Montagnes horse breed
Source: Genet Sel Evol. 2024 Jul 10;56:53. doi: 10.1186/s12711-024-00922-6 (PMC11238448; doi:10.1186/s12711-024-00922-6)
Supplement: Supplementary file 6 — Additional file 6: Table S5. Runs of homozygosity segments shared by more than 50% of Franches-Montagnes. Table S5 presents the runs of homozygosity segments shared by more than 50% of Franches-Montagnes including the length and position along the chromosomes. The annotated genes within the segments are also reported. [file 12711_2024_922_MOESM6_ESM.pdf]

Table S5 : Runs of homozygosity segments shared by over 50% of Franches-Montagnes.

Chr.	Length (Mb)	Begin	End	Annotated genes
1	0.51	30971234	31479608	*HPSE2, HPS1, PYROXD2, R3HCC1L, LOXL4*
2	0.53	27876021	28462821	*PTAFR, EYA3, XKR8, SMPDL3B, RPA2, THEMIS2, PPP1R8, STX12, FAM76A, IFI6, FGR, AHDC1*
3	0.91	120437951	121351057	*NKX1-1, UVSSA, MAEA, CTBP1, SPON2, RNF212, FGFRL1, IDUA, SLC26A1, DGKQ, TMEM175, GAK, CPLX1, PCGF3, SLC49A3, MYL5, ATP5ME, PDE6B, PIGG*
11	2.32	23273876	25588903	*FBXO47, LASP1, RPL23, CWC25, PIP4K2B, PSMB3, PCGF2, CISD3, MLLT6, EPOP, SRCIN1, ARHGAP23, SOCS7, GPR179, MRPL45, NPEPPS, KPNB1, TBKBP1, TBX21, OSBPL7, MRPL10, LRRC46, SCRN2, SP6, SP2, PNPO, PRR15L, CDK5RAP3, COPZ2, NFE2L1, CBX1, SNX11, SKAP1, HOXB1, HOXB2, HOXB3, HOXB5, HOXB6, HOXB7, HOXB8, HOXB9, HOXB13, TTLL6, CALCOCO2, ATP5MC1, UBE2Z, SNF8, GIP, IGF2BP1, B4GALNT2, GNGT2, ABI3, PHOSPHO1, ZNF652, PHB, NGFR, NXPH3, SPOP*
11	0.28	26151832	26426827	*LRRC59, ACSF2, CHAD, RSAD1, MYCBPAP, EPN3, SPATA20, CACNA1G, ABCC3, ANKRD40, LUC7L3*
11	0.03	26485440	26516656	*WFIKKN2*
11	0.26	31311231	31570264	*NOG, DGKE*
