# Supplementary material for: Using high-density SNP data to unravel the origin of the Franches-Montagnes horse breed
Source: Genet Sel Evol. 2024 Jul 10;56:53. doi: 10.1186/s12711-024-00922-6 (PMC11238448; doi:10.1186/s12711-024-00922-6)
Supplement: Supplementary file 7 — Additional file 7: Table S6. Gene ontology analysis for the Purebred Arabians. Table S6 presents the results from the gene ontology analysis for the genes present in the runs of homozygosity islands for the Purebred Arabians. [file 12711_2024_922_MOESM7_ESM.pdf]

Table S6: Gene ontology analysis for the Purebred Arabians

Term	Bonferroni adjusted p-value	N genes	Genes
**GO biological process**			
intermediate filament organization (GO:0045109)	7.29E-09	11	*KRT24, KRT25, KRT12, KRT28, KRT10B, KRT27, KRT23, KRT20, KRT10A, KRT39, KRT26*
intermediate filament cytoskeleton organization (GO:0045104)	9.17E-09	12	*KRT24, KRT25, KRT12, KRT28, KRT10B, KRT27, KRT23, KRT20, KRT10A, KRT39, CSNK1A1, KRT26*
intermediate filament-based process (GO:0045103)	1.07E-08	12	*KRT24, KRT25, KRT12, KRT28, KRT10B, KRT27, KRT23, KRT20, KRT10A, KRT39, CSNK1A1, KRT26*
epithelial cell differentiation (GO:0030855)	1.02E-03	15	*KRT24, KRT25, THRA, INTU, KRT12, PLK4, KRT28, RARA, KRT10B, KRT27, KRT23, KRT20, KRT10A, KRT39, KRT26*
**GO molecular function**			
-	-	-	-
**GO cellular component**			
intermediate filament (GO:0005882)	2.32E-09	13	*KRT24, KRT25, KRT12, KRT28, KRT10B, KRT27, KRT23, KRT20, KRT10A, KRT39, CSNK1A1, KRT222, KRT26*
intermediate filament cytoskeleton (GO:0045111)	2.63E-08	13	*KRT24, KRT25, KRT12, KRT28, KRT10B, KRT27, KRT23, KRT20, KRT10A, KRT39, CSNK1A1, KRT222, KRT26*
polymeric cytoskeletal fiber (GO:0099513)	8.97E-05	18	*KRT24, KRT25, MAPRE1, CENPE, KIF3B, KRT12, KRT28, KRT10B, KRT27, KRT23, KRT20, KRT10A, KRT39, TUBB3, CSNK1A1, KRT222, GAS8, KRT26*
supramolecular fiber (GO:0099512)	1.35E-03	19	*KRT24, KRT25, MAPRE1, CENPE, KIF3B, KRT12, KRT28, KRT10B, KRT27, KRT23, KRT20, KRT10A, KRT39, CASQ2, TUBB3, CSNK1A1, KRT222, GAS8, KRT26*
supramolecular polymer (GO:0099081)	1.48E-03	19	*KRT24, KRT25, MAPRE1, CENPE, KIF3B, KRT12, KRT28, KRT10B, KRT27, KRT23, KRT20, KRT10A, KRT39, CASQ2, TUBB3, CSNK1A1, KRT222, GAS8, KRT26*
supramolecular complex (GO:0099080)	1.06E-03	23	*KRT24, SSB, KRT25, MAPRE1, CENPE, KIF3B, LARP1B, KRT12, STK31, KRT28, KRT10B, KRT27, KRT23, KRT20, CASC3, KRT10A, KRT39, CASQ2, TUBB3, CSNK1A1, KRT222, GAS8, KRT26*
