# Supplementary material for: Using high-density SNP data to unravel the origin of the Franches-Montagnes horse breed
Source: Genet Sel Evol. 2024 Jul 10;56:53. doi: 10.1186/s12711-024-00922-6 (PMC11238448; doi:10.1186/s12711-024-00922-6)
Supplement: Supplementary file 8 — Additional file 8: Table S7. Gene ontology analysis for the Shagya Arabians. Table S7 presents the results from the gene ontology analysis for the genes present in the runs of homozygosity islands for the Shagya Arabians. [file 12711_2024_922_MOESM8_ESM.pdf]

Table S7: Gene ontology analysis for the Shagya Arabians

Term	Bonferroni adjusted p-value	N genes	Genes
**GO biological process**			
regulation of transcription initiation by RNA polymerase II (GO:0060260)	3.38E-02	5	*MED24, THRA, TAF12, MED13, MED1*
**GO molecular function**			
transcription factor binding (GO:0008134)	6.53E-03	14	*RNF25, MED24, THRA, STK36, DGKQ, CTBP1, RARA, TAF12, SMARCE1, MTDH, MED13, CREB1, MED1, NR1D1*
binding (GO:0005488)	4.39E-03	128	*CDK12, CLIP1, UBR3, STAC2, CCR7, SSB, DDX55, KNTC1, RNF25, MTMR4, IKZF3, PPP1R8, IL31, MED24, CCR3, AK3, TEX14, LOC100068349, TOP2A, RHOF, FGR, RPL19, GEM, THRA, TRNAU1AP, RB1CC1, DNAJC8, POP1, WIPF2, LAPTM4B, PDP1, SPATA6L, NKX1-1, FGFRL1, YTHDF2, IGFBP4, DGKE, NOG, MSI2, CSF3, LPO, GAK, STK36, DGKQ, ATP5IF1, CTBP1, RARA, TAF12, CDC37L1, ALKAL1, METTL5, KRT27, AKAP1, PDE6B, OPRD1, RILPL1, SMARCE1, MYO3B, SESN2, CDC6, DNAH10, MAEA, PXDNL, MATN2, XXYLT1, PRKAG3, TSPOAP1, STK3, GLIS3, CCRL2, CASC3, BRIP1, GTF2H3, MSL1, NBAS, PPM1E, CCR2, TJP1, HPD, MPO, LRRC43, HSF5, RAD51C, RNF212, SETD1B, SLC1A1, GSDMA, MTDH, MED13, CREB1, RILPL2, RAB42, TRIM25, GSDMB, LTF, SNRNP35, RAD54B, MED1, MYL5, STX12, UVSSA, SUPT4H1, METTL8, PHACTR4, ATP6V0A2, ESRP1, ZNF142, CDH17, NR1D1, ZCCHC8, RBM12B, MLXIP, LTF, EIF2B1, EYA3, GMEB1, BCS1L, SMPDL3B, SPON2, ARL5C, RPL30, COIL, RNF43, CYP27A1, LOC100147364, RPA2, CPLX1, ST18*
**GO cellular component**			
intermediate filament (GO:0005882)	4.15E-02	7	*KRT24, KRT25, KRT28, KRT10B, KRT27, KRT222, KRT26*
organelle (GO:0043226)	4.33E-04	128	*CDK12, CLIP1, KRT24, CCR7, SSB, DDX55, KNTC1, RNF25, IKZF3, PPP1R8, MED24, AK3, TEX14, LOC100068349, TOP2A, FGR, SLC49A3, KRT25, RPL19, TCTN2, THRA, TRNAU1AP, RB1CC1, DNAJC8, ERC2, POP1, TMEM67, IFI6, LAPTM4B, PDP1, SPATA6L, SNTG1, NKX1-1, KRT28, YTHDF2, ORMDL3, DIABLO, PIGG, DGKE, MSI2, GAK, STK36, DGKQ, ATP5IF1, CTBP1, RARA, TAF12, TMED2, KMT5A, METTL5, KRT10B, TMEM175, KRT27, AKAP1, PDE6B, RCL1, OPRD1, RILPL1, SMARCE1, MYO3B, SESN2, THEMIS2, CDC6, B3GNT4, DNAH10, MAEA, XXYLT1, PRKAG3, TSPOAP1, DPY19L4, STK3, GLIS3, CASC3, BRIP1, GTF2H3, TRIQK, MSL1, NBAS, PPM1E, PCGF3, TJP1, AHDC1, HPD, MPO, LRRC43, HSF5, RAD51C, RNF212, SETD1B, SLC1A1, MTDH, MED13, CREB1, RILPL2, VPS33A, LTF, SNRNP35, RAD54B, MED1, STX12, UVSSA, SUPT4H1, METTL8, ATP6V0A2, ESRP1, ZNF142, VIRMA, NR1D1, ZCCHC8, KRT222, RBM12B, MLXIP, LTF, EYA3, DCAF17, FAM76A, GMEB1, BCS1L, ARL5C, KRT26, RPL30, COIL, CYP27A1, LOC100147364, RPA2, ST18, BCL7A, PSMD9*
intracellular organelle (GO:0043229)	1.47E-03	124	*CDK12, CLIP1, KRT24, CCR7, SSB, DDX55, KNTC1, RNF25, IKZF3, PPP1R8, MED24, AK3, TEX14, LOC100068349, TOP2A, FGR, SLC49A3, KRT25, RPL19, THRA, TRNAU1AP, RB1CC1, DNAJC8, ERC2, POP1, IFI6, LAPTM4B, PDP1, SNTG1, NKX1-1, KRT28, YTHDF2, ORMDL3, DIABLO, PIGG, DGKE, MSI2, GAK, STK36, DGKQ, ATP5IF1, CTBP1, RARA, TAF12, TMED2, KMT5A, METTL5, KRT10B, TMEM175, KRT27, AKAP1, RCL1, OPRD1, RILPL1, SMARCE1, MYO3B, SESN2, THEMIS2, CDC6, B3GNT4, DNAH10, MAEA, XXYLT1, PRKAG3, TSPOAP1, DPY19L4, STK3, GLIS3, CASC3, BRIP1, GTF2H3, TRIQK, MSL1, NBAS, PPM1E, PCGF3, TJP1, AHDC1, HPD, MPO, LRRC43, HSF5, RAD51C, RNF212, SETD1B, SLC1A1, MTDH, MED13, CREB1, RILPL2, VPS33A, LTF, SNRNP35, RAD54B, MED1, STX12, UVSSA, SUPT4H1, METTL8, ATP6V0A2, ESRP1, ZNF142, VIRMA, NR1D1, ZCCHC8, KRT222, RBM12B, MLXIP, LTF, EYA3, DCAF17, FAM76A, GMEB1, BCS1L, ARL5C, KRT26, RPL30, COIL, CYP27A1, LOC100147364, RPA2, ST18, BCL7A, PSMD9*
membrane-bounded organelle (GO:0043227)	2.52E-02	113	*CDK12, CLIP1, CCR7, SSB, DDX55, RNF25, IKZF3, PPP1R8, MED24, AK3, LOC100068349, TOP2A, SLC49A3, TCTN2, THRA, TRNAU1AP, RB1CC1, DNAJC8, POP1, TMEM67, IFI6, LAPTM4B, PDP1, SPATA6L, NKX1-1, YTHDF2, ORMDL3, DIABLO, PIGG, DGKE, MSI2, GAK, STK36, DGKQ, ATP5IF1, CTBP1, RARA, TAF12, TMED2, KMT5A, METTL5, TMEM175, AKAP1, PDE6B, RCL1, OPRD1, RILPL1, SMARCE1, SESN2, THEMIS2, CDC6, B3GNT4, DNAH10, MAEA, XXYLT1, PRKAG3, TSPOAP1, DPY19L4, STK3, GLIS3, CASC3, BRIP1, GTF2H3, TRIQK,*

			NBAS, PPM1E, PCGF3, TJP1, AHDC1, HPD, MPO, LRRC43, HSF5, RAD51C, RNF212, SETD1B, SLC1A1, MTDH, MED13, CREB1, RILPL2, VPS33A, LTF, SNRNP35, RAD54B, MED1, STX12, UVSSA, SUPT4H1, METTL8, ATP6V0A2, ESRP1, ZNF142, VIRMA, NR1D1, ZCCHC8, RBM12B, MLXIP, LTF, EYA3, DCAF17, FAM76A, GMEB1, BCS1L, ARL5C, RPL30, COIL, CYP27A1, LOC100147364, RPA2, ST18, BCL7A, PSMD9
intracellular anatomical structure (GO:0005622)	5.79E-05	142	*CDK12, CLIP1, KRT24, UBR3, CCR7, SSB, DDX55, KNTC1, RNF25, MTMR4, IKZF3, PPP1R8, MED24, CCR3, AK3, TEX14, LOC100068349, TOP2A, RHOF, FGR, SLC49A3, KRT25, RPL19, THRA, TRNAU1AP, RB1CC1, DNAJC8, ERC2, POP1, IFI6, LAPTM4B, PDP1, SNTG1, RCC1, NKX1-1, KRT28, YTHDF2, ORMDL3, DIABLO, PIGG, DGKE, MSI2, LPO, GAK, STK36, DGKQ, ATP5IF1, CTBP1, RARA, TAF12, TMED2, KMT5A, CDC37L1, METTL5, KRT10B, TMEM175, KRT27, AKAP1, RCL1, OPRD1, RILPL1, SMARCE1, MYO3B, SESN2, PSMD3, THEMIS2, CDC6, B3GNT4, DNAH10, MAEA, XXYLT1, PRKAG3, TSPOAP1, DPY19L4, STK3, GLIS3, CCRL2, CASC3, BRIP1, GTF2H3, TRIQK, MSL1, NBAS, PPM1E, PCGF3, CCR2, TJP1, AHDC1, HPD, MPO, LRRC43, HSF5, RAD51C, RNF212, SETD1B, SLC1A1, GSDMA, MTDH, MED13, CREB1, RILPL2, METTL21A, VPS33A, TRIM25, LTF, SNRNP35, RAD54B, MED1, MYL5, STX12, UVSSA, SUPT4H1, METTL8, PHACTR4, ATP6V0A2, ESRP1, ZNF142, VIRMA, NR1D1, PCMTD1, ZCCHC8, KRT222, RBM12B, MLXIP, LTF, EIF2B1, EYA3, DCAF17, FAM76A, GMEB1, BCS1L, ARL5C, KRT26, RPL30, COIL, CYP27A1, LOC100147364, RPA2, CPLX1, ST18, BCL7A, PSMD9*
cellular anatomical entity (GO:0110165)	3.36E-03	173	*CDK12, CLIP1, KRT24, UBR3, STAC2, CCR7, SSB, DDX55, KNTC1, COL22A1, RNF25, MTMR4, IKZF3, PPP1R8, IL31, MED24, CCR3, AK3, TEX14, LOC100068349, TOP2A, RHOF, FGR, SLC49A3, OPCML, KRT25, RPL19, GEM, TCTN2, THRA, TRNAU1AP, RB1CC1, DNAJC8, ERC2, POP1, TMEM67, IFI6, LAPTM4B, PDP1, SPATA6L, SNTG1, RCC1, NKX1-1, KRT28, FGFRL1, YTHDF2, IGFBP4, ORMDL3, DIABLO, PIGG, DGKE, NOG, MSI2, CSF3, LPO, GAK, STK36, DGKQ, ATP5IF1, CTBP1, RARA, TAF12, TMED2, KMT5A, CDC37L1, ALKAL1, METTL5, KRT10B, TMEM175, KRT27, AKAP1, PDE6B, RCL1, OPRD1, RILPL1, SMARCE1, MYO3B, SESN2, PSMD3, THEMIS2, CDC6, B3GNT4, DNAH10, MAEA, PXDNL, SLC26A1, MATN2, XXYLT1, PRKAG3, TSPOAP1, DPY19L4, STK3, GLIS3, CCRL2, CASC3, BRIP1, GTF2H3, TRIQK, MSL1, NBAS, CACNB1, PPM1E, PCGF3, CCR2, TNS4, TJP1, LRRC3C, AHDC1, HPD, MPO, LRRC43, HSF5, RAD51C, RNF212, SETD1B, XKR8, SLC1A1, GSDMA, MTDH, MED13, CREB1, RILPL2, METTL21A, VPS33A, TRIM25, GSDMB, LTF, SNRNP35, RAD54B, MED1, MYL5, STX12, UVSSA, SUPT4H1, METTL8, PHACTR4, ATP6V0A2, ESRP1, ZNF142, VIRMA, NTM, CDH17, NR1D1, PCMTD1, ZCCHC8, KRT222, RBM12B, MLXIP, LTF, EIF2B1, EYA3, DCAF17, FAM76A, GMEB1, BCS1L, KCNS2, SMPDL3B, NIPAL2, SPON2, ARL5C, KRT26, RPL30, RAPGEFL1, COIL, RNF43, CYP27A1, LOC100147364, PLXDC1, RPA2, CPLX1, ST18, BCL7A, PSMD9*
