# Supplementary material for: Using high-density SNP data to unravel the origin of the Franches-Montagnes horse breed
Source: Genet Sel Evol. 2024 Jul 10;56:53. doi: 10.1186/s12711-024-00922-6 (PMC11238448; doi:10.1186/s12711-024-00922-6)
Supplement: Supplementary file 9 — Additional file 9: Table S8. Gene ontology analysis for the Thoroughbred. Table S8 presents the results from the gene ontology analysis for the genes present in the runs of homozygosity islands for the Thoroughbred. [file 12711_2024_922_MOESM9_ESM.pdf]

Table S8 : Gene ontology analysis for the Thoroughbred

Term	Bonferroni adjusted p-value	N genes	Genes
**GO cellular component**			
plasma membrane region (GO:0098590)	3.96E-02	27	*ADGRL1, EPS8L3, GRID1, ADCY1, SLC26A2, KCNA2, MTTP, FAP, PDE6A, CIB1, DPP4, AQP6, SEMA4B, LIMA1, GABRB3, KRT8, SLC26A11, PRKACA, AQP2, GABRG3, SHROOM1, SLC22A5, SLC16A1, SYNDIG1, AQP2, RACGAP1, AQP5*
