# Supplementary material for: Using high-density SNP data to unravel the origin of the Franches-Montagnes horse breed
Source: Genet Sel Evol. 2024 Jul 10;56:53. doi: 10.1186/s12711-024-00922-6 (PMC11238448; doi:10.1186/s12711-024-00922-6)
Supplement: Supplementary file 10 — Additional file 10: Table S9. Gene ontology analysis for the old-type Franches-Montagnes. Table S9 presents the results from the gene ontology analysis for the genes present in the runs of homozygosity islands for the old-type Franches-Montagnes. [file 12711_2024_922_MOESM10_ESM.pdf]

Table S9 : Gene ontology analysis for the old-type Franches-Montagnes population

Term	Bonferroni adjusted p-value	N genes	Genes
**GO biological process**			
rhombomere development (GO:0021546)	1.06E-02	3	*HOXB2, HOXB1, HOXB3*
embryonic skeletal system morphogenesis (GO:0048704)	3.31E-02	5	*HOXB9, HOXB2, HOXB1, HOXB5, HOXB3*
regulation of transcription by RNA polymerase II (GO:0006357)	9.02E-03	25	*CDK5RAP3, HOXB9, HOXB7, SP6, NFE2L1, HOXB8, NKX1-1, SKAP1, ZNF652, DGKQ, CTBP1, HOXB2, HOXB13, HOXB1, PCGF3, HOXB5, CBX1, HOXB3, HOXB6, MLLT6, SP2, KAT7, RPL23, PCGF, TBX21*
