# Supplementary material for: Using high-density SNP data to unravel the origin of the Franches-Montagnes horse breed
Source: Genet Sel Evol. 2024 Jul 10;56:53. doi: 10.1186/s12711-024-00922-6 (PMC11238448; doi:10.1186/s12711-024-00922-6)
Supplement: Supplementary file 11 — Additional file 11: Table S10. Gene ontology analysis for the Franches-Montagnes. Table 10 presents the results from the gene ontology analysis for the genes present in the runs of homozygosity islands for the Franches-Montagnes. [file 12711_2024_922_MOESM11_ESM.pdf]

Table S10 : Gene ontology analysis for the Franches-Montagnes population

Term	Bonferroni adjusted p-value	N genes	Genes
**GO biological process**			
rhombomere development (GO:0021546)	1.69E-02	3	*HOXB2, HOXB1, HOXB3*
embryonic skeletal system morphogenesis (GO:0048704)	2.93E-03	6	*HOXB9, NOG, HOXB2, HOXB1, HOXB5, HOXB3*
embryonic skeletal system development (GO:0048706)	1.29E-02	6	*HOXB9, NOG, HOXB2, HOXB1, HOXB5, HOXB3*
skeletal system morphogenesis (GO:0048705)	5.09E-03	8	*FGR, HOXB9, NOG, HOXB2, HOXB1, HOXB5, HOXB3, PHOSPHO1*
**GO cellular component**			
nucleoplasm (GO:0005654)	4.80E-03	27	*PPP1R8, HOXB9, NFE2L1, EPN3, SKAP1, DGKE, DGKQ, CTBP1, CALCOCO2, UBE2Z, SPOP, HOXB2, IGF2BP1, HOXB13, HOXB1, HOXB5, MRPL10, CBX1, HOXB3, STX12, UVSSA, SP2, EYA3, FAM76A, LUC7L3, RPL23, PIP4K2B*
nuclear lumen (GO:0031981)	1.11E-03	31	*PPP1R8, CDK5RAP3, HOXB9, NFE2L1, EPN3, SKAP1, DGKE, DGKQ, CTBP1, CALCOCO2, UBE2Z, SPOP, MAEA, HOXB2, IGF2BP1, HOXB13, HOXB1, HOXB5, RNF212, MRPL10, CBX1, HOXB3, STX12, UVSSA, SP2, EYA3, FAM76A, LUC7L3, RPL23, RPA2, PIP4K2B*
organelle lumen (GO:0043233)	5.46E-03	32	*PPP1R8, CDK5RAP3, HOXB9, NFE2L1, EPN3, SKAP1, DGKE, DGKQ, CTBP1, CALCOCO2, UBE2Z, SPOP, MAEA, HOXB2, IGF2BP1, HOXB13, PYROXD2, HOXB1, HOXB5, RNF212, MRPL10, CBX1, HOXB3, STX12, UVSSA, SP2, EYA3, FAM76A, LUC7L3, RPL23, RPA2, PIP4K2B*
intracellular organelle lumen (GO:0070013)	5.46E-03	32	*PPP1R8, CDK5RAP3, HOXB9, NFE2L1, EPN3, SKAP1, DGKE, DGKQ, CTBP1, CALCOCO2, UBE2Z, SPOP, MAEA, HOXB2, IGF2BP1, HOXB13, PYROXD2, HOXB1, HOXB5, RNF212, MRPL10, CBX1, HOXB3, STX12, UVSSA, SP2, EYA3, FAM76A, LUC7L3, RPL23, RPA2, PIP4K2B*
membrane-enclosed lumen (GO:0031974)	5.46E-03	32	*PPP1R8, CDK5RAP3, HOXB9, NFE2L1, EPN3, SKAP1, DGKE, DGKQ, CTBP1, CALCOCO2, UBE2Z, SPOP, MAEA, HOXB2, IGF2BP1, HOXB13, PYROXD2, HOXB1, HOXB5, RNF212, MRPL10, CBX1, HOXB3, STX12, UVSSA, SP2, EYA3, FAM76A, LUC7L3, RPL23, RPA2, PIP4K2B*
